# Diamond-Like Carbon Depositing on the Surface of Polylactide Membrane for Prevention of Adhesion Formation During Tendon Repair

**DOI:** 10.1007/s40820-024-01392-7

**Published:** 2024-04-30

**Authors:** Yao Xiao, Zaijin Tao, Yufeng Ju, Xiaolu Huang, Xinshu Zhang, Xiaonan Liu, Pavel A. Volotovski, Chao Huang, Hongqi Chen, Yaozhong Zhang, Shen Liu

**Affiliations:** 1 Department of Orthopaedics, Shanghai Jiao Tong University School of Medicine Affiliated Sixth People’s Hospital, 600 Yishan Rd, Shanghai, 200233 People’s Republic of China; 2 Shanghai Tongji Hospital, 389 Xincun Rd, Shanghai, 200065 People’s Republic of China; 3 Key Laboratory for Thin Film and Microfabrication of Ministry of Education, Research Institute of Micro/Nano Science and Technology, Shanghai Jiao Tong University, Shanghai, 200240 People’s Republic of China; 4 Orthopedic Trauma Department, Belarus Republic Scientific and Practical Center for Traumatology and Orthopedics, Kizhevatova str., 60/4, 220024 Minsk, Belarus; 5 Shanghai Haohai Biological Technology Limited Liability Company, 1386 Hongqiao Rd, Shanghai, 200336 People’s Republic of China; 6 Department of General Surgery, Shanghai Jiao Tong University School of Medicine Affiliated Sixth People’s Hospital, 600 Yishan Rd, Shanghai, 200233 People’s Republic of China; 7 Shanghai Key Laboratory for High Temperature Materials and Precision Forming, School of Materials Science and Engineering, Shanghai Jiao Tong University, Shanghai, 200240 People’s Republic of China

**Keywords:** Diamond-like carbon, Reactive oxygen species scavenging, Foreign body reaction, Biodegradation, Antioxidant, Peritendinous adhesion

## Abstract

Post-traumatic peritendinous adhesion presents a significant challenge in clinical medicine. This study proposes the use of diamond-like carbon (DLC) deposited on polylactic acid (PLA) membranes as a biophysical mechanism for anti-adhesion barrier to encase ruptured tendons in tendon-injured rats. The results indicate that PLA/DLC composite membrane exhibits more efficient anti-adhesion effect than PLA membrane, with histological score decreasing from 3.12 ± 0.27 to 2.20 ± 0.22 and anti-adhesion effectiveness increasing from 21.61% to 44.72%. Mechanistically, the abundant C=O bond functional groups on the surface of DLC can reduce reactive oxygen species level effectively; thus, the phosphorylation of NF-κB and M1 polarization of macrophages are inhibited. Consequently, excessive inflammatory response augmented by M1 macrophage-originated cytokines including interleukin-6 (IL-6), interleukin-1*β* (IL-1*β*), and tumor necrosis factor-*α* (TNF-*α*) is largely reduced. For biocompatibility evaluation, PLA/DLC membrane is slowly absorbed within tissue and displays prolonged barrier effects compared to traditional PLA membranes. Further studies show the DLC depositing decelerates the release of degradation product lactic acid and its induction of macrophage M2 polarization by interfering esterase and PLA ester bonds, which further delays the fibrosis process. It was found that the PLA/DLC membrane possess an efficient biophysical mechanism for treatment of peritendinous adhesion.

**Supplementary Information:**

The online version contains supplementary material available at 10.1007/s40820-024-01392-7.

## Highlights

- The anti-adhesion effect of polylactic acid (PLA) membrane with diamond-like carbon (DLC) depositing is 44.72%, enhanced by 23.11% compared to PLA.
- DLC deposited on PLA membranes has been shown to effectively reduce the levels of reactive oxygen species, leading to a decrease in the expression of pro-inflammatory cytokines within peritendinous adhesion tissue.
- DLC decelerates PLA biodegradation and lactic production, which reduces the number of CD68^+^CD206^+^ macrophages within peritendinous adhesion tissue.

**Supplementary Information:**

The online version contains supplementary material available at 10.1007/s40820-024-01392-7.

## Graphical abstract

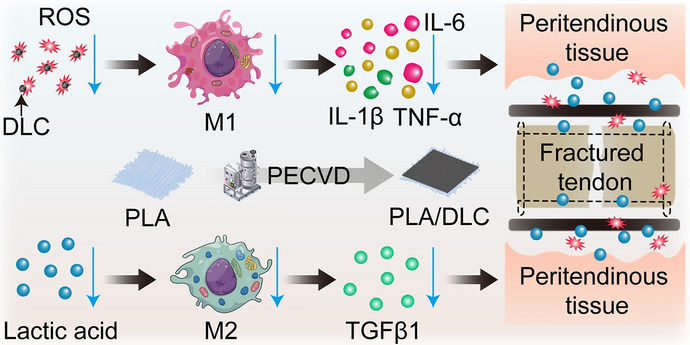

## Introduction

Peritendinous adhesion formation is one of the most common and challenging complications during the healing process following tendon injuries and tendon surgeries [1, 2]. Until now, approximately 30%–40% of patients suffer from peritendinous adhesion to the surrounding tissues after tendon surgeries [3]. Pathologically, the granulation tissue invades into injury site from surrounding tissues to promote tendon healing as an exogenous repair [4]. However, the excessive exogenous repair leads to abnormal adhesions to the surrounding tissue that limits tendon sliding, excursion, and range of motion, eventually restricting joint motion [5]. The adhesions also increase the risk of a secondary tendon rupture because of the forceful training and mobilization [6]. Furthermore, the decreased range of motion caused by adhesions can prolong rehabilitation and necessitate reoperation, reducing patients’ quality of life and exacerbating psycho-socioeconomic problems [7, 8].

Several methods have been developed to prevent peritendinous adhesion, including surgical tenolysis, systemic and local drug administration, postoperative physical therapy, and the use of physical barriers or their combination [9, 10]. Among these, the electrospun fibrous polylactic acid (PLA) membrane is a favored anti-adhesion strategy, serving as a physical barrier. It can inhibit myofibroblast proliferation and invasion into the repaired tendon site while allowing nutrient exchange through a microporous structure [1, 11]. In addition, PLA-based micro-nanofibers are also used for anti-scarring [12]. PLA and its derivatives are selected due to excellent biocompatibility, biodegradability, and bioresorbability. However, their widespread application as anti-adhesion membranes, artificial stents, and wound dressings can lead to foreign body reactions (FBR) upon PLA membrane implantation, resulting in local inflammation that undermines its anti-adhesion effect [11, 13]. In particular, when the implantation of the PLA membrane activates the host immune system, it is prone to recruiting neutrophils and macrophages to the membrane surface, inducing an inflammatory microenvironment [14]. The infiltrated macrophages secrete pro-inflammatory cytokines like interleukin-6 (IL-6), interleukin-1*β* (IL-1*β*), and tumor necrosis factor-*α* (TNF-*α*), which recruit fibroblasts and promote their differentiation into myofibroblasts, leading to collagen deposition, fibrotic encapsulation, and adhesion [15–17]. The previous study has revealed that electrospun fibrous PLA membranes can promote macrophage infiltration, inflammation, and granuloma formation [18], thereby aggravating FBR through NF-κB mediated M1 polarization of macrophages [17]. On the other hand, the biodegradation of the PLA membrane and the released by-products exaggerate peritendinous adhesion formation, compromising its anti-adhesion efficiency. To be specific, the hydrolysis and esterification of implanted PLA membranes in vivo upregulate the biodegradation products like lactic acid in surrounding tissues, leading to severe postoperative fibrosis, thrombosis, and endothelial-mesenchymal transition [19, 20]. It was found that the degraded PLA nanofibers can promote M2 macrophage polarization through the STAT6 pathway in peritendinous adhesion [11]. From these two perspectives, PLA membrane degradation and foreign body-induced macrophage polarization dramatically weaken the anti-adhesion efficacy of PLA membranes. Therefore, modification of the PLA membrane is essential to prevent inflammation induced by foreign bodies, delay biodegradation, and enhance anti-adhesion efficacy.

With a large number of patients worldwide suffering from acute wounds, wound healing has become a pressing and complex issue [21]. Effective wound care is essential to prevent infection, alleviate pain, facilitate the healing process, and minimize scar formation, driving rapid advancements in biomaterials within tissue engineering [22, 23]. The high-level reactive oxygen species (ROS) in microenvironments induced by surgical trauma activates inflammatory response and is involved in tissue repair [24, 25]. Based on the well-established physiology of wound healing, ROS plays a significant role in the healing process and accompanies hyperplasia [26]. From this perspective, antioxidant-related biomaterials have attracted the attention of researchers. Series of creative redox regulatory biomaterials are developed for tissue repair, including manganese-based antioxidase-inspired biocatalysts networks [27], catalase-mimetic artificial biocatalysts with Ru catalytic centers for ROS elimination [28], cascade and ultrafast artificial antioxidases [24], ROS-catalytic metalloenzyme-mimics with atomic metal centers [29], and some other antioxidase-like nanobiocatalysts with ultrafast and reversible redox-centers [30, 31]. Especially, barrier membrane with ROS scavenging property offers significant advantages in prevention of postoperative adhesion [32].

As for tendon injury, excessive ROS production at the lesion sites exacerbates oxidative stress and inflammation [33], which may induce fibrosis and unexpected tissue adhesion [34, 35]. Generally, the ROS level shows a significant increase in the injured tendon within 72 h due to acute trauma. Meanwhile, peritendinous adhesion gradually emerges and deteriorates [36]. Considering oxidative stress as a therapeutic target for tissue fibrosis [37–39], quercetin and phlorotannin can be used as antioxidants to reduce peritendinous adhesion effectively [40, 41]. As for FBR, Neutrophils are the first responders who adhered to the protein layer surrounding the implant within minutes of implantation, and they release ROS to promote the inflammatory response [42]. Accordingly, the ROS also acts as an essential mediator of FBR-induced inflammation. Given the above facts, ROS scavenging is a feasible and effective strategy for preventing tendon adhesion information and reducing the FBR of membrane implantation.

Carbon-based nanomaterials constituted crystalline diamond cores within amorphous carbon layers, with numerous functional groups of oxygen on the surface [43]. Carbon-based nanomaterials are suitable for biological and medical applications, especially for drug delivery and biomedical imaging, owing to their nontoxicity, inherent biocompatibility, superior colloidal dispersibility, and high chemical resistance [44–46]. Previous studies have demonstrated carbon-based nanomaterials' radical scavenging ability and anti-inflammatory properties due to large specific surface area and numerous surface functional groups [47–50]. Thomas et al. observed a significant reduction in the expression of inflammatory markers such as Tnf-*α*, Il-1*β*, and chemokine Ccl2 in RAW264.7 macrophages exposed to carbon-based nanomaterials, indicating their anti-inflammatory potential [51]. Additionally, Alawdi et al. demonstrated that carbon-based nanomaterials mitigated memory deficits in the rat model of Alzheimer's disease by suppressing oxidative stress and downregulating the expression of pro-inflammatory cytokines TNF-*α* and IL-6 mediated by NF-κB and STAT3 [52]. From this perspective, carbon-based nanomaterials can be utilized as anti-adhesion biomaterials.

This study used diamond-like carbon (DLC) as a carbon-based nanomaterial and deposited on the PLA membrane. Then, the PLA/DLC membrane was implanted at the site of tendon injury in a rat model of peritendinous adhesion. The results suggested that the DLC-depositing PLA membrane exerted antioxidative capacity both in vitro and in vivo, further attenuating foreign body-induced inflammation and effectively preventing peritendinous adhesion information. Meanwhile, DLC hindered PLA membrane degradation and lactic acid release. The antioxidant and anti-degradation capabilities of PLA/DLC highlight their significant potential as anti-adhesion barriers. In addition, this study provides a novel therapeutic approach for preventing peritendinous adhesion and expands the scope of application for carbon-based nanomaterials in osteopathic medicine.

## Methods

### Preparation of DLC and PLA/DLC Membrane

The PLA membrane was synthesized as previously described [15]. Briefly, dichloromethane (sigma, USA) and N, N-dimethylformamide (sigma, USA) were mixed at 7:3 to prepare a mixed solvent. PLA (sigma, USA) was stirred in the mixed solvent at a 90 mg mL^−1^ concentration. Then, the PLA solution was subjected to the electrospinning machine (Ucalery, Beijing, China) at a 1.2 mL h^−1^ rate. The ejected PLA nanofibers from the needle tip were collected on aluminum foil. Subsequently, the PLA membranes were dried in ovens and collected for use.

**Fig. 1:**
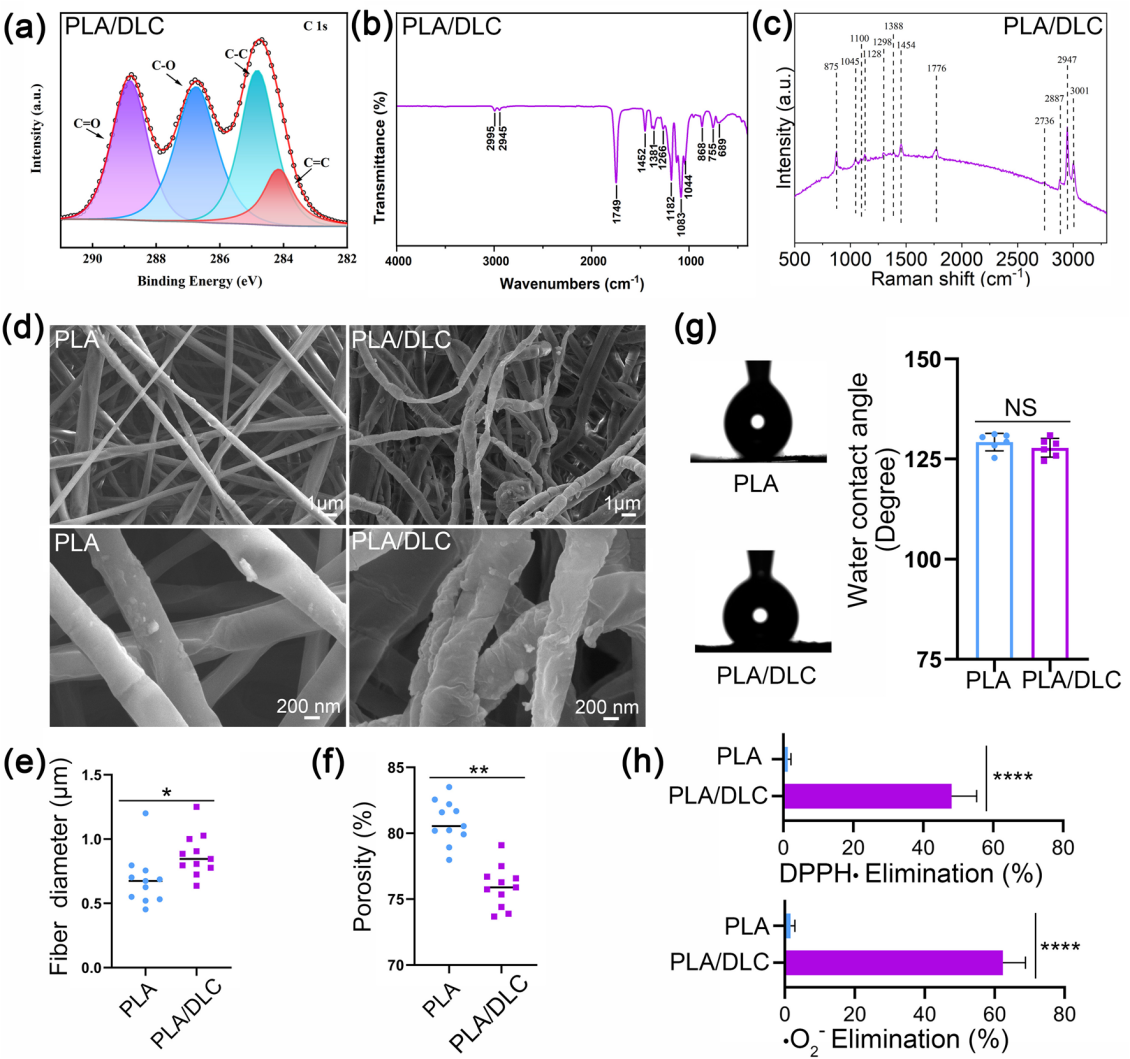
Characterization of the PLA/DLC membranes. **a** XPS spectra of PLA/DLC. **b** FTIR spectrum. **c** Raman spectroscopy. **d** SEM micrographs, **e** fiber diameter and **f** porosity. **g** Water contact angle image and quantifying. **h** DPPH· and ·O_2_^−^ scavenging. Data represent independent experiments, and all data are presented as mean ± SD; *NS* non-significant, *P* > 0.05; *P < 0.05; **P < 0.01; ****P < 0.0001

The PLA/DLC membrane was synthesized by depositing DLC film with the plasma-enhanced chemical vapor deposition (PECVD) method described before [53]. The deposition substrates PLA membranes were placed in a chemical vapor deposition chamber following high evacuation. Then, argon was introduced into the vacuum chamber to produce high-density Ar plasma by 600 W plasma excitation unit to clean and preheat the PLA membrane for 10 min. The Ar flow rate was set to 150 mL min^−1^, and the chamber pressure was set to 1.5 Pa. The reaction gas methane was introduced with a 50 mL min^−1^ flow rate to deposit DLC film on the PLA membrane surface for 15 min. Under a growth rate of 300 nm h^−1^, the thickness of the deposited DLC film was 10 nm. During this depositing process, the Ar flow rate was maintained at 150 mL min^−1^, and the chamber pressure was set to 15 Pa. The scanning electron microscope images of PLA and PLA/ DLC membranes are showed in Fig. 1d.

### Characterizations

The element composition and chemical valence of the membrane were determined by an X-ray photoelectron spectroscope (XPS, Thermo ESCALAB 250XI, USA) with 1486.6 eV Al K*α* excitation source, and the results were analyzed by the C 1*s* peak at 285 eV. The positions and intensities of the absorption peaks of PLA/DLC membrane were recorded using Fourier transform infrared (FTIR) Tracer-100 Spectrometer (Shimadzu) and confocal micro-Raman spectrometer (InVia, Renishaw, UK), respectively. The scanning electron microscopy (SEM) images were captured to evaluate the membrane morphology with VEGA 3 TESCAN (Czech). The average fiber diameter and porosity of PLA and PLA/DLC membranes were measured using ImageJ 1.53 (USA) SEM images. The hydrophilism of the membranes was evaluated with water contact angles on a contact angle analyzer (DSA25S, Data Physics Corporation).

The methods and principles of radical scavenging test in vitro were well described previously [25, 29]. The 1,1-diphenyl-2-picrylhydrazyl radical (DPPH ·) scavenging property was assayed with a DPPH Free Radical Scavenging Capacity Assay Kit (Solarbio, China) following the manufacturer’s instructions. The ·O_2_^−^ scavenging property was test with a Superoxide Anion Activity Content Assay Kit (Solarbio, China) following the manufacturer’s instructions.

### In vitro Investigation

#### PLA Degradation and Lactic Acid Release

Dried PLA or PLA/DLC membranes were cut into disks of 20 mm diameters, and the initial weight was recorded. Sliced membranes were immersed in 25 mL of pH 8.0 Tris buffer containing 10% esterase concentration and 10 mM calcium chloride to imitate the biodegradation environment of tendon injury. Then, the reaction system was sealed and stored at 37 °C. Degraded weighted loss was calculated with dry weight as 0, 1, 4, 6, 10, 14, 21, and 28 days, and a weight-time curve was drawn.

The degraded reaction solution was obtained for measuring lactic acid content at each time point, and lactic acid concentration was measured following the instructions of Amplite™ Colorimetric L-Lactate Assay Kit (Cat.13815, AAT Bioquest, USA). The concentration of released lactic acid was calculated, and a content-time curve was drawn.

#### Cell Culture and Treatment

The mouse embryonic fibroblast-derived cells NIH/3T3 and the mouse macrophage-derived cells RAW264.7 were purchased from the Shanghai Institute of Cell Biology (China). NIH/3T3 and RAW264.7 cells were routinely cultured in high-glucose DMEM medium (Hyclone, Logan, USA) supplemented with 10% fetal bovine serum (Gibco, USA), 100 units/mL of penicillin (Gibco™) and 100 μg mL^−1^ of streptomycin (Gibco™) at 37 °C and in a humidified atmosphere containing 5% CO_2._ As for the cell inoculation on the membrane, PLA or PLA/DLC membranes were cut appropriately and pressed into the well with a steel ring to avoid floating. The membranes were immersed with 75% alcohol for 1 h under ultraviolet irradiation. After discarding the alcohol and washing the membrane with PBS three times, NIH/3T3 or RAW264.7 cells were cultured on the membrane for further experiments.

#### Cell Proliferation, Live/Dead Cell Staining, and Cell Adhesion Assay

The effect of PLA and PLA/DLC membranes was assessed on NIH/3T3 cell proliferation. Cells were incubated with PLA or PLA/DLC membranes for 1, 3, and 5 days. Cell proliferation was detected using a CCK8 Assay Kit (C0038, Beyotime Institute of Biotechnology, China) following the manufacturer’s instructions. After incubation with CCK8 reaction reagent for 1 h, the cell supernatant was divided equally into 96 well plates, and the absorbance was read at 450 nm. After 1 and 3 days of incubation on PLA or PLA/DLC membranes, the viability of NIH/3T3 cells was determined using a Calcein/PI Live/Dead Viability/Cytotoxicity Assay Kit (C2015M, Beyotime Institute of Biotechnology, China), and the dead/live cell rate was assessed. For cell adhesion assay, after incubation for 1 and 3 days, the cytoskeletal arrangement of NIH/3T3 cells was observed by actin staining with Phalloidin Kit (Invitrogen™, USA) following manufacturer’s instructions. Images were taken using a fluorescence microscope (Olympus, 178 BX43, Japan).

#### Cell Oxidative Stress Assessment

To measure cytosolic ROS level, NIH/3T3 cells were cultured on PLA or PLA/ DLC membranes in 24-well and incubated with H_2_O_2_ (100 μM) for 24 h. According to the manufacturer’s instructions, cytosolic ROS was measured using a ROS Assay Kit (S0033S, Beyotime Institute of Biotechnology, China). Stained cells were visualized and captured by a fluorescence microscope (Olympus, 178 BX43, Japan). Fluorescence intensity was assessed with the public domain software ImageJ 1.53 (USA).

#### Macrophage Polarization

RAW264.7 cells were cultured on PLA or PLA/DLC membranes in 24-well grew for 1 day to form a confluent monolayer and incubated with H_2_O_2_ (100 μM) for 24 h. Cells were fixed with 4% paraformaldehyde for 20 min, permeabilized with 0.5% Triton × 100 in PBS, blocked with 5% BSA, and then incubated with Arg-1 (1:100 dilution, Arginase-1, Rabbit mAb #93,668, CST, USA) and iNOS (1:100 dilution, Rabbit mAb #13,120, CST, USA) antibodies at 4 °C overnight. The nucleus was stained with DAPI. Images were captured using a fluorescence microscope (Olympus, BX43, Japan). Fluorescence intensity was evaluated with the public domain software ImageJ 1.53 (USA). In addition, the polarization of RAW264.7 cells was also evaluated by flow cytometry with FITC-anti CD80 (104,706, biolegend, USA) antibodies [54].

For quantitative RT-PCR, the total RNA was extracted from RAW264.7 cells using FastPure Total RNA Isolation Kit (RC101, Vazyme, Nanjing, China). Subsequently, cDNA was synthesized from RNA with an HiScript III 1st Strand cDNA Synthesis Kit (R312-01, Vazyme, Nanjing, China). The mRNA sample was analyzed with qPCR. Taq Pro Universal SYBR qPCR Master Mix (Q712-02, Vazyme, Nanjing, China) on ABI prism 7500 Sequence Detection System (Applied Biosystems) was used for qPCR following the manufacture's instructions. The relative expression of genes was calculated by 2^–ΔΔCT^ method. qRT-PCR primer sequences are shown in Table S1.

To determine the inflammatory cytokine levels of RAW264.7 cells, mouse IL-6 ELISA Kit (ABN-KA2272, Abnova, Taiwan, Chian) and Mouse TNF-*α* ELISA Kit (BE69212, IBL-America) were used to determine the concentration of IL-6 and TNF-*α* in the supernatant of RAW264.7 cells, respectively.

### In Vivo Investigation

#### Preparation of Rat Achilles Tendon Adhesion Model

Eight-week-old male Sprague–Dawley (SD) rats were purchased from Shanghai Laboratory Animal Company (Shanghai, China). All experimental procedures and protocols followed the Guidelines for the Care and Use of Laboratory Animals: Eighth Edition (ISBN-10: 0-309-15396-4). All the protocols were approved by the Institutional Review Committee of Shanghai Jiao Tong University (SYXK (Hu) 2018-0028). All rats were housed in SPF-level conditions under 12-h dark/light cycles at 22 ± 2 °C with a humidity of 55 ± 5% and free access to food and water. Rats were anesthetized with isoflurane, and the surgical site was shaved and disinfected. The Achilles tendon was exposed by longitudinal shin incision and then the tendon was transected and repaired by the Kessler suture method with 5–0 sterile sutures. The PLA and PLA/DLC membranes were cut with 0.5 × 1.0 cm^2^ pieces and wrapped around the injury site. For the Sham group, the PLA and PLA/DLC membranes were directly wrapped around the tendon without transection and repair. After surgery, rats were kept for sample collection for 7, 14, and 28 days (*n* = 12).

#### Gross Evaluation

Before anatomical assessment, rats were euthanized, and the surgical site was exposed. The inflammatory and infective ulcers of the tendon were observed, and images were captured. The degree of peritendinous adhesion was evaluated using an adhesion scoring system described in our previous study [4, 11]. The adhesion scoring was classified into 5 grades: 1, no obvious adhesion at the surgical site; 2, limited adhesion tissue but can be bluntly separated; 3, less than 50% of the adhesion tissue that needed sharp separation; 4, nearly 51%–97.5% of the adhesion tissue which needed sharp separation; 5, more than 97.5% severe adhesion tissue that required sharp separation.

#### Histological Assessment

After the surgery, the hind limbs were fixed with 4% paraformaldehyde for 1 day after perfusion, followed by decalcification in 10% EDTA with shaking for 4 weeks. The fixed tissue was dehydrated in graded ethanol, embedded in paraffin, and then sliced into 5 μm sections with a microtome (Leica RM2235, Germany). The sections were subjected to hematoxylin & eosin (H&E) staining, Masson’s staining, and immunohistochemical staining of Collagen III (1:300 dilution, sc-271249, Santa Cruz, USA) and IL-1*β* (1:400 dilution, ab283818, Abcam, UK). The degree of adhesion of the injured tendon was assessed using a 1–5 grade histological scoring system [4]. The sections were also subjected to immunofluorescence assay with rat anti-CD68 antibody (1:200 dilution, ab283654, Abcam, UK), anti-iNOS (1:200 dilution, ab178945, Abcam, UK), and anti-CD206 (1:200 dilution, ab64693, Abcam, UK). All stained sections were visualized using a fluorescence microscope (BX43, Olympus, Japan). Images were analyzed by the public domain software ImageJ 1.53.

#### Western Blotting

The total protein of peritendinous tissues was isolated using cell lysis buffer (P0013, Beyotime Institute of Biotechnology, China) as per manufacturer’s instructions. Western blotting was performed following the standard protocol [17]. Antibodies against HO-1 (1:1000 dilution, ab68477, Abcam, UK), Nrf2 (1:1000 dilution, AF0639, Affinity Biosciences, USA), NF-κB (1:1000 dilution, Rabbit mAb #8242, CST, USA), p-NF-κB (Ser536) (1:1000 dilution, Rabbit mAb #3033, CST, USA), and β-Actin (1:2000 dilution, Rabbit mAb #4970, CST, USA) were used for Western blotting.

#### Detection of Tissue Oxidation Products

Seven days after surgery, rat peritendinous tissues were homogenized in cold 1 × PBS, followed by two freeze–thaw cycles. Then, the homogenates were centrifuged at 5000 × g, 4 °C for 5 min. The supernatants were aliquotted and assayed immediately. The tissue content of MDA was measured using a Lipid Peroxidation MDA Assay Kit (S0131S, Beyotime Institute of Biotechnology, China) and following manufacturer's instructions. The concentration of nucleic acid oxidative product 8-OHdG was determined using a Rat 8-OHdG ELISA Kit (CSB-E10526r, Cusabio, USA) following manufacturer's instructions. The protein oxidative product 3-NT level was assayed using a rat 3-NT ELISA Kit (F16301, Westang, Shanghai, China).

#### Inflammatory Cytokine Level in Peritendinous Tissue

The Rat IL-6 ELISA Kit (BE69158, IBL-America), Rat TNF-*α* ELISA Kit (A1010A0320, BioTNT, Shanghai, China), and Rat IL-1*β* ELISA Kit (61-I1BRT-E01, ALPCO, USA) were used to detect IL-6, TNF-*α*, and IL-1*β* levels in rat peritendinous tissues.

### Statistical Analysis

All experiments were performed at least in triplicate, and results are presented as mean ± SEM. ANOVA analysis with Tukey’s posthoc test was used to compare multiple groups. *P* values < 0.05 were considered statistically significant. Statistical analyses were conducted using SPSS (v22.0, IBM Inc., USA), and figures were generated by Prism v8.2.1 (GraphPad Software Inc., USA).

## Results

### PLA/DLC Suppressed ROS Production and Alleviated Oxidative Stress without Affecting the Fibroblast Proliferation and Adhesion

The C 1*s* core-level spectrum deconvoluted with Gaussian curves is displayed in Fig. 1a. The characteristic peak at 284.2 eV was corresponded to C=C (*sp*^2^ carbon), the peak at 284.8 eV to the C–C (*sp*^3^ carbon), the peak at 286.7 eV to the C-O, and the peak at 288.8 eV to the C=O, respectively. These results were consistent with previous study [53]. Specifically, the peak at 288.8 eV was mainly resulted from the C=O dangling bonds on DLC, which have been proved to have a potential for free radical scavenging [55]. PLA/DLC displayed an efficient DPPH· and ·O_2_^−^ elimination property (47.3% and 63.4% within 1 h, respectively (Fig. 1h)) although failed to catalyze H_2_O_2_ to produce O_2_ (data not shown), demonstrating that radical scavenging property of DLC might attribute to the stabilization of radical rather than CAT-like activity. FTIR spectrum of PLA/DLC membrane showed strong peaks at 2995 and 2945 cm^−1^ corresponding to C-H stretch (Fig. 1b), which was related to biocompatibility. Raman spectra identified strong peaks at 1128 and 1776 cm^−1^ on PLA/DLC, which corresponding to C–O and C=O, respectively (Fig. 1c). The membrane morphology of PLA and PLA/DLC membranes are shown in Fig. 1d. The SEM images revealed that the post-deposited DLC film was tightly attached to the outer layer of the PLA fibers, indicating a good contact had been formed. The PLA fibers were slippy, round, regular, and straight, with a diameter of 0.68 ± 0.20 μm. In contrast, the PLA/DLC fibers were bent, irregular, and uneven with a diameter of 0.88 ± 0.17 μm (Fig. 1e). Moreover, the porosity of the membrane was significantly decreased (Fig. 1f) resulted from thickened and tortuous fiber when deposited DLC film. The water contact angles showed no difference between PLA and PLA/DLC membrane (Fig. 1g).

**Fig. 2:**
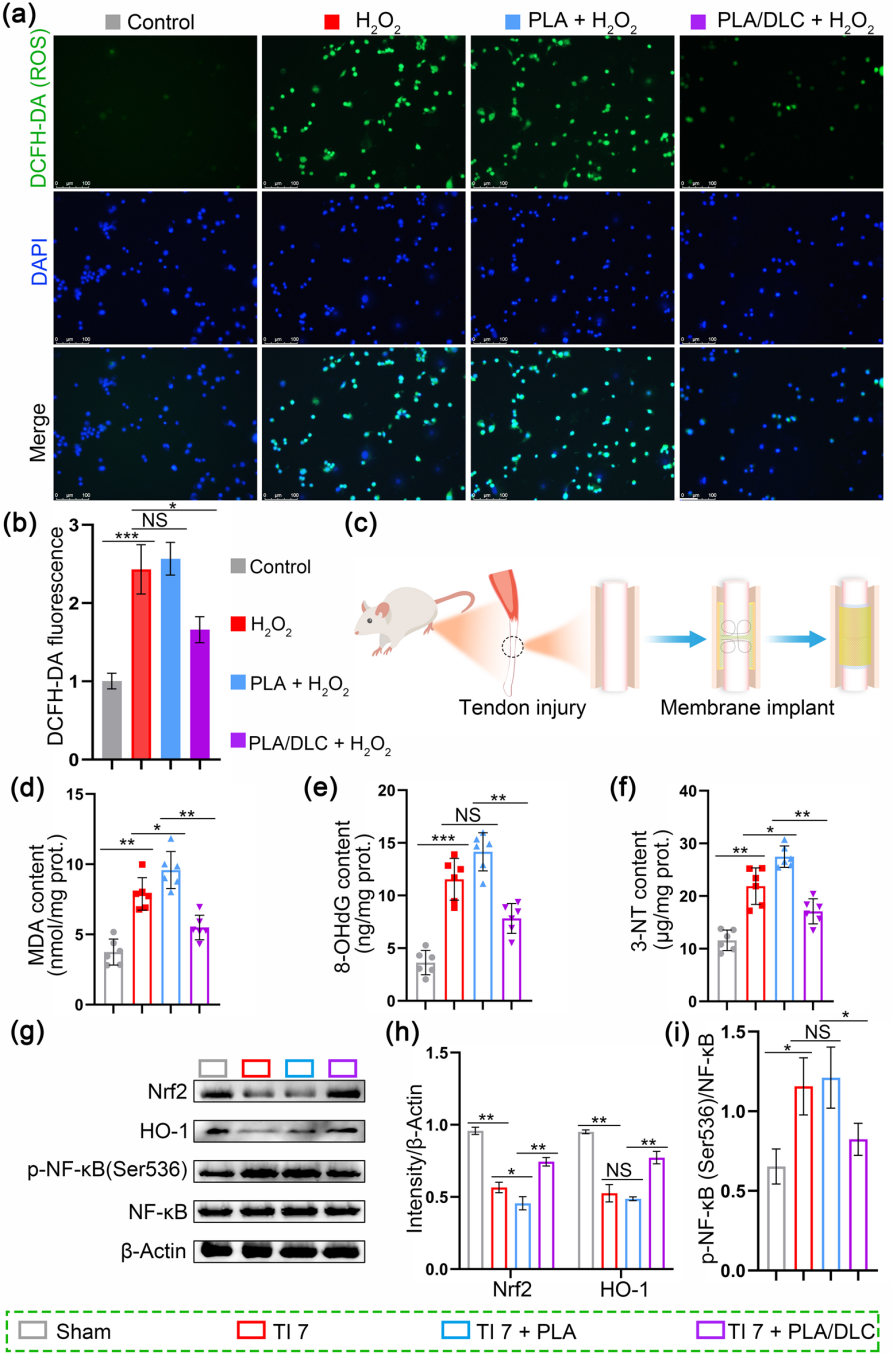
DLC suppress ROS production and oxidative stress both in vitro and in vivo. **a** Detection of cell ROS level on different membranes using ROS probes and **b** statistical analysis. **c** Diagram of animal experiment. **d**, **f** Content of MDA, 8-OHdG, and 3-NT in peritendinous tissues after 7 days of injury was examined by assay kit respectively. **g** Representative bands of Nrf2, HO-1, p-NF-κB were measured by Western blotting. **h** Nrf2 and HO-1 expression normalized to *β*-actin expression. **i** p-NF-κB expression normalized to NF-κB expression. Data represent independent experiments, and all data are presented as mean ± SD; *NS* non-significant, *P* > 0.05; *P < 0.05; **P < 0.01; ***P < 0.001, ****P < 0.0001

Besides the free radical scavenging test in vitro, DLC’s antioxidant property was also assessed at the cellular level and in animal models. Initially, a cellular lesion model was first established by treating NIH/3T3 cells with H_2_O_2_. DCFH-DA fluorescence probe was employed to assess the antioxidant property of the DLC-deposited PLA membrane. Higher concentrations of intracellular ROS were indicated by stronger green fluorescence intensity. Exposure of cells to H_2_O_2_ remarkably augmented cytosolic ROS production, as evidenced by the green fluorescence intensity enhancement (Fig. 2a, b). Similarly, cells cultured on the PLA membrane exhibited elevated ROS levels. However, when cultured on the DLC-deposited PLA membrane, notably lower ROS levels were observed, likely due to the ROS scavenging ability of DLC (Fig. 2a, b). Correspondingly, PLA/DLC treatment notably reduced the levels of MDA (lipid peroxidation product), 8-OHdG (biomarker of oxidative DNA damage), and 3-NT (protein oxidative product) in H_2_O_2_-stimulated cells (Fig. S1a-c). All of these primary results indicated a radical scavenging potential of DLC.

To investigate the antioxidant property of DLC in vivo, we established a rat model of Achilles tendon injury (TI) as depicted in Fig. 2c, and the PLA membrane and PLA/ DLC membrane were implanted surrounding the tendon. The tendon was intact in the sham group. After 7 days of tendon injury, we measured the peroxidation products of lipids, proteins, and nucleic acids in peritendinous tissues to evaluate the levels of oxidative stress. Specifically, the lipid peroxidation marker MDA was quantified in the peritendinous tissues. The result showed that the MDA concentration was markedly increased in the injured tendon compared to the sham group. In contrast, this concentration was notably reduced following the implantation of PLA/DLC membranes (Fig. 2d). Additionally, 8-OHdG is a biomarker of oxidative DNA damage [56]. PLA/DLC implantation potently inhibited the injury induced by 8-OHdG in peritendinous tissues (Fig. 2e). The tissue level of protein oxidative product 3-NT was significantly elevated in peritendinous tissues, which was reversed by PLA/DLC implantation (Fig. 2f). Moreover, the MDA and 3-NT content in PLA group were significantly higher than those in tendon injury group, respectively (Fig. 2d, f), suggesting a higher level of ROS induced by PLA membrane implantation. These findings indicate that PLA/DLC implantation markedly suppressed injury-induced oxidative stress in peritendinous tissues, compared with PLA membrane implantation, and DLC reduced the ROS level induced by PLA implantation (Fig. 2d-f).

Furthermore, the inhibitory effects of DLC on oxidative stress were also confirmed in peritendinous tissues. As displayed in Fig. 2g, h, the expression of antioxidant proteins Nrf2 and HO-1 were significantly decreased in peritendinous tissues after tendon injury, and PLA/DLC membrane implantation markedly upregulated their expression. In contrast, PLA membrane implantation showed a lower Nrf2 level than the tendon injury group (Fig. S2). In addition, tendon injury markedly induced NF-κB hyperphosphorylation at Ser536 in peritendinous tissues, and significantly, PLA/DLC membrane implantation effectively reversed NF-κB hyperphosphorylation compared with PLA membrane (Fig. 2g, i).

**Fig. 3:**
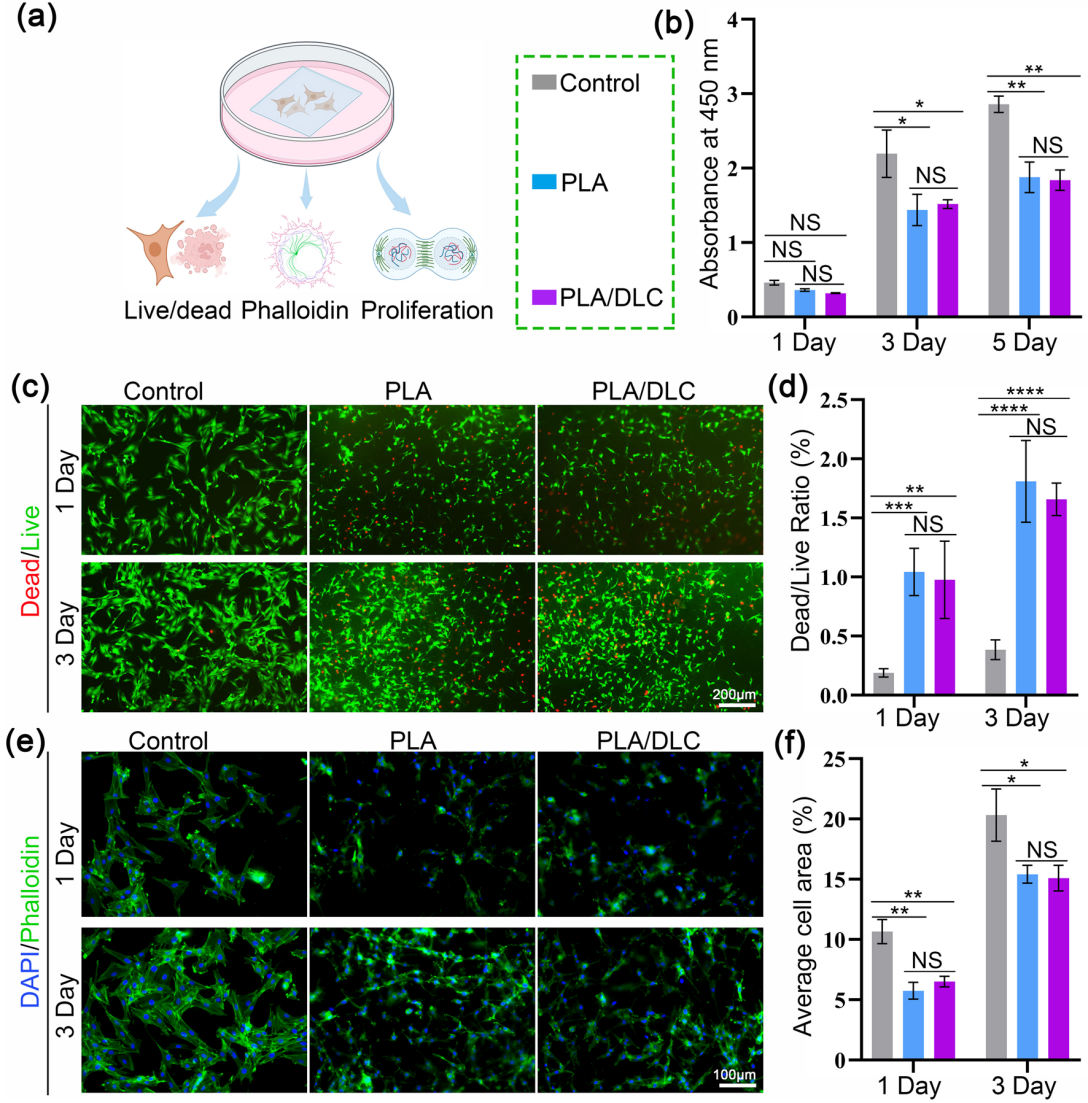
DLC do not alter the proliferation and adhesion of fibroblasts in vitro. **a** Fibroblasts live/dead, proliferation, and adhesion analysis in vitro. **b** Proliferation of fibroblasts cultured on different membranes at days 1, 3, and 5 were measured using CCK8 kits. **c** Live/dead staining of fibroblasts cultured on different membranes for 1 and 3 days, and **d** Dead/live cell rate. Red: dead cells; green: live cells. **e** Adhesion analysis of fibroblasts cultured on different membranes at days 1 and 3, and **f** cellular arrangement area. Data represent independent experiments, and all data are given as mean ± SD; *NS* non-significant, *P* > 0.05; *P < 0.05; **P < 0.01; ***P < 0.001, ****P < 0.0001

To evaluate the effect of DLC on cell proliferation and adhesion, a series of assays, including live/dead cell staining, phalloidin staining, and cell viability tests, were performed after culturing NIN/3T3 fibroblasts on PLA membrane or PLA/DLC membrane (Fig. 3a). The control group included cells directly cultured in the plates. Firstly, the proliferation of NIN/3T3 fibroblasts at 1, 3, and 5 days after culturing was assessed using a CCK8 kit. As shown in Fig. 3b, the absorbance of the three groups (Control, PLA, and PLA/DLC) had no profound difference at 1 day. However, the absorbance of PLA and PLA/DLC groups significantly decreased at 3 and 5 days compared with the control group. Nevertheless, when compared with cells cultured on the PLA membrane, the absorbance of the PLA/DLC group showed no significant change at 3 and 5 days. Live/dead cell staining consistently showed similar results (Figs. 3c, d and S3). The ratio of dead cells in the PLA and PLA/DLC groups significantly increased at 1 and 3 days, respectively, compared to the control group. However, there was no difference observed between PLA and PLA/DLC groups at 1 and 3 days. Moreover, the adhesion ability of fibroblasts was determined by cell-cytoskeleton staining with phalloidin at 1 and 3 days, and no noticeable difference in cell area was observed between PLA and PLA/DLC groups (Figs. 3e, f and S4).

Oxidative stress after tissue injury notably affects fibrosis and adhesion [36]. ROS plays a significant role in peritendinous adhesion via mediating fibrogenesis, and several studies have revealed that downregulating ROS can effectively prevent peritendinous adhesion [36, 40, 57, 58]. In this study, DLC exhibited antioxidant capacity both in vitro and in vivo. DLC decreased the ROS level in H_2_O_2_-induced NIH/3T3 cells, and alleviated oxidative stress in the inflammation stage (7 days) of tendon injury (Fig. 2). As it is already acknowledged that carbon nanoparticles have good electron-transfer acceleration capability, implying a potent candidate for manipulation of ROS in biomedical field [55]. The radical scavenging property of DLC has been proved in the DPPH· reaction system [43, 47], and DLC further displayed ROS-reduction effect in cells and in mice [49, 59]. These studies strongly evidenced the antioxidant capacity of PLA/DLC membrane that prevents adhesion formation in our study. The antioxidant property of DLC was attributed to the reduction of ROS. The mechanism might be based on the unsatisfied bonds and numerous C=O bonds on the DLC surface, and these unsaturated bonds allowed the redox reactions to occur with ROS. Thus, the ROS scavenging ability of DLC might have originated from the abundant functional groups on its surface, where the ROS could be stabilized by the rearrangement of functional groups among themselves or by the resonance of unpaired electrons [43, 59]. In a related matter, another study also implied that the C=O bonds were significantly related to the antibacterial ability of the DLC [60]. On the other hand, hydrogen-terminated DLC revealed negative electron affinity that could yield facile electron emission, thus scavenging the ROS [61].

Cumulatively, these results indicated that DLC effectively suppressed excessive intracellular ROS production and oxidative stress both in vitro and in vivo, highlighting their potent antioxidant properties. However, DLC did not affect cell proliferation and adhesion when deposited on the PLA membrane.

### PLA/DLC Showed Anti-Inflammatory Properties in Vitro

**Fig. 4:**
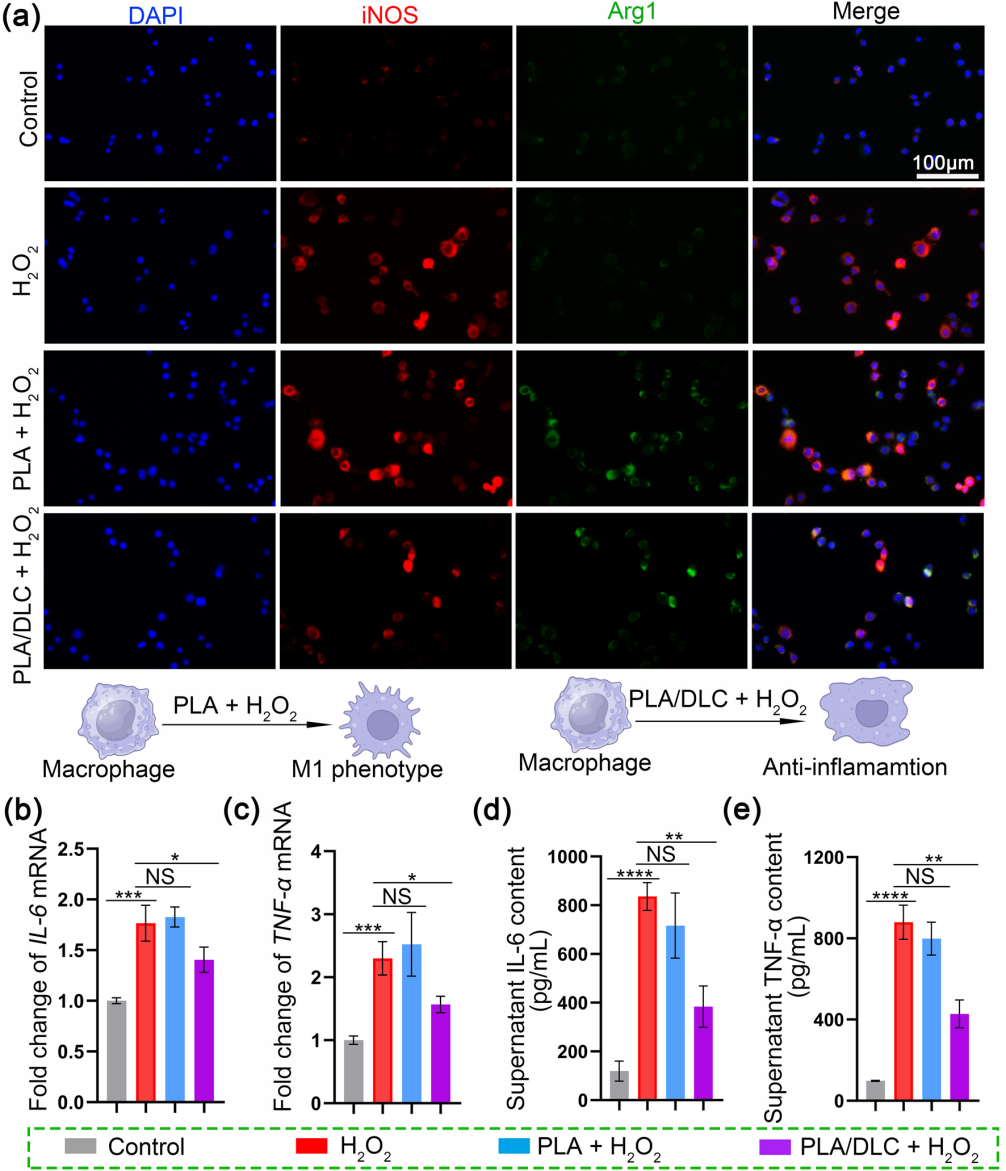
DLC showed anti-inflammatory properties in vitro. The H_2_O_2_ group was established in vivo. **a** iNOS/Arg-1 fluorescence double-staining was used to analyze the anti-inflammatory properties of PLA/DLC. **b**, **c** The mRNA expression levels of IL-6 and TNF-*α* were detected by real-time PCR. **d**, **e** The concentrations of TNF-*α* and IL-6 in cell supernatant were measured by ELISA. Data represent independent experiments, and all data are given as mean ± SD; *NS* non-significant, *P* > 0.05; *P < 0.05; **P < 0.01; ***P < 0.001, ****P < 0.0001

The effect of DLC on macrophage polarization was measured on RAW264.7 cells, which were cultured on PLA and PLA/DLC membranes. In the control group, cells were directly cultured on plates. H_2_O_2_ was used to simulate the inflammatory microenvironment with oxidative properties [62]. The expression of iNOS (red) was significantly increased after H_2_O_2_ stimulation (Fig. 4a), indicating that cells were differentiated into the M1 phenotype. PLA/DLC remarkably downregulated iNOS expression under H_2_O_2_ stimulation, whereas the PLA membrane had no such effect. In addition, flow cytometry results also proved that DLC can decrease the M1 macrophages (Fig. S5). Correspondingly, H_2_O_2_ dramatically upregulated the mRNA levels of pro-inflammatory genes *IL-6* and *TNF-α* and enhanced the secretion of IL-6 and TNF-*α* (Fig. 4b-e). Interestingly, PLA/DLC inhibited H_2_O_2_-induced upregulation and secretion of pro-inflammatory factors IL-6 and TNF-*α*. In contrast, PLA did not affect the expression and secretion of IL-6 and TNF-*α* (Fig. 4b-de). These results demonstrate that DLC can prevent M1 polarization of macrophages and dampen the pro-inflammatory response.

### PLA/DLC Mitigated Inflammation Response and Abnormal Hyperplasia Caused by PLA Membrane Implantation

**Fig. 5:**
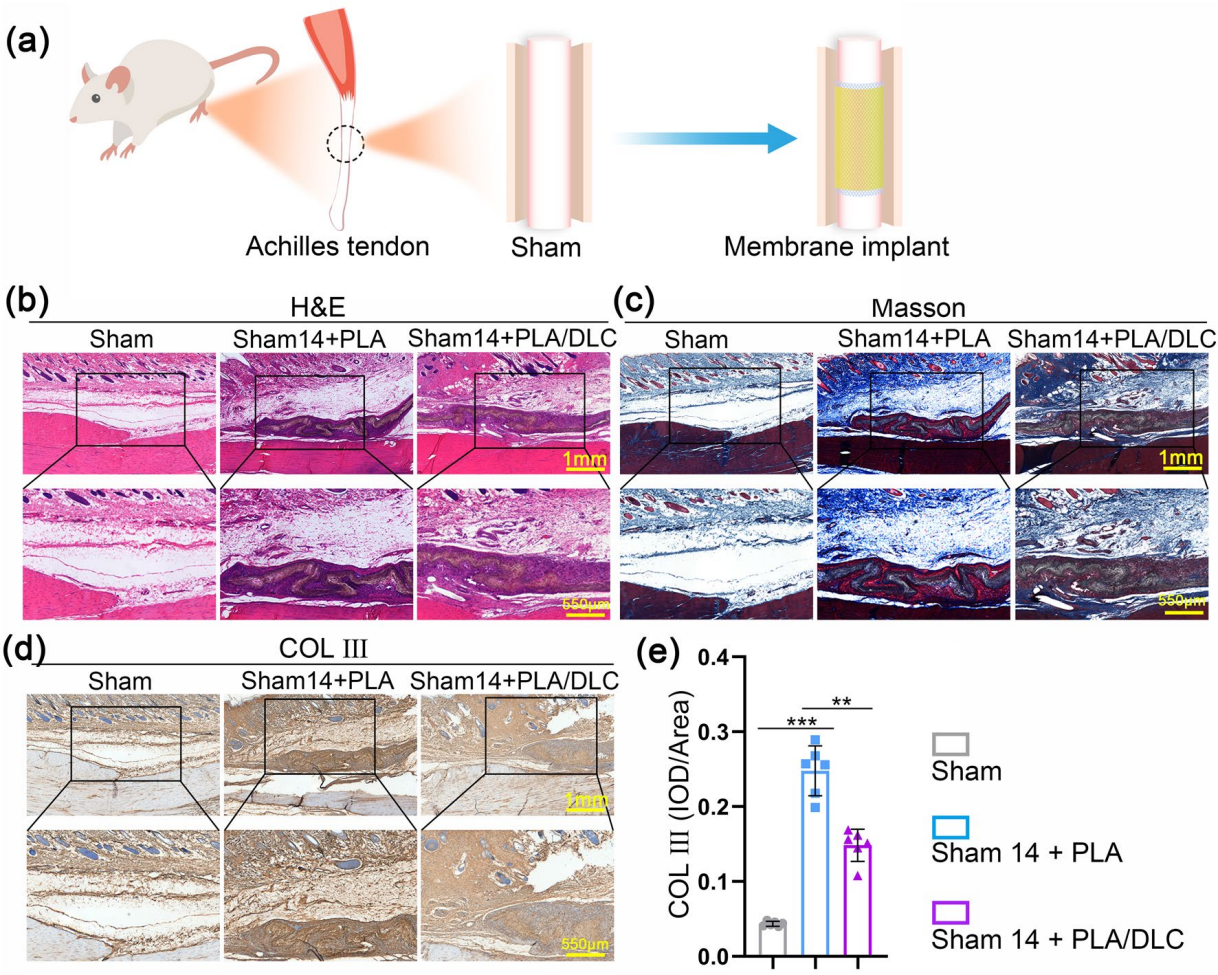
DLC inhibited inflammatory infiltration and abnormal hyperplasia induced by PLA membrane implantation. **a** Diagram of animal experiment. **b** H&E staining and **c** Masson trichrome staining of normal tendon wrapped by PLA and PLA/DLC for 14 d. **d** Immunohistochemical staining for COL III of Sham group, Sham + PLA group, and Sham + PLA/DLC group at 14 d. **e** Optical density of COL III was measured in the peritendinous tissues. Data represent independent experiments, and all data are presented as mean ± SD; *NS* non-significant, *P* > 0.05; *P < 0.05; **P < 0.01; ***P < 0.001, ****P < 0.0001

To assess the difference in FBR between PLA membrane and PLA/DLC membrane in vivo, we implanted the membranes around the intact tendon in rats for 14 days (Fig. 5a). No biological toxicity was observed when the membrane was implanted around rat tendon (Fig. S6). Histological staining was then used to assess implantation-induced inflammatory response and abnormal hyperplasia. As shown in Fig. 5b, c, H&E staining and Masson staining indicated that PLA membrane implantation induced severe inflammatory infiltration and abnormal hyperplasia. PLA/DLC implantation alleviated the inflammatory infiltration and abnormal hyperplasia compared with PLA implantation. Compared with the Masson staining, immunohistochemical staining of COL III also displayed that DLC coating inhibited PLA implantation-induced abnormal hyperplasia (Fig. 5d, e). These results revealed that DLC alleviate FBR and inhibited PLA implantation-induced inflammatory infiltration and abnormal hyperplasia.

**Fig. 6:**
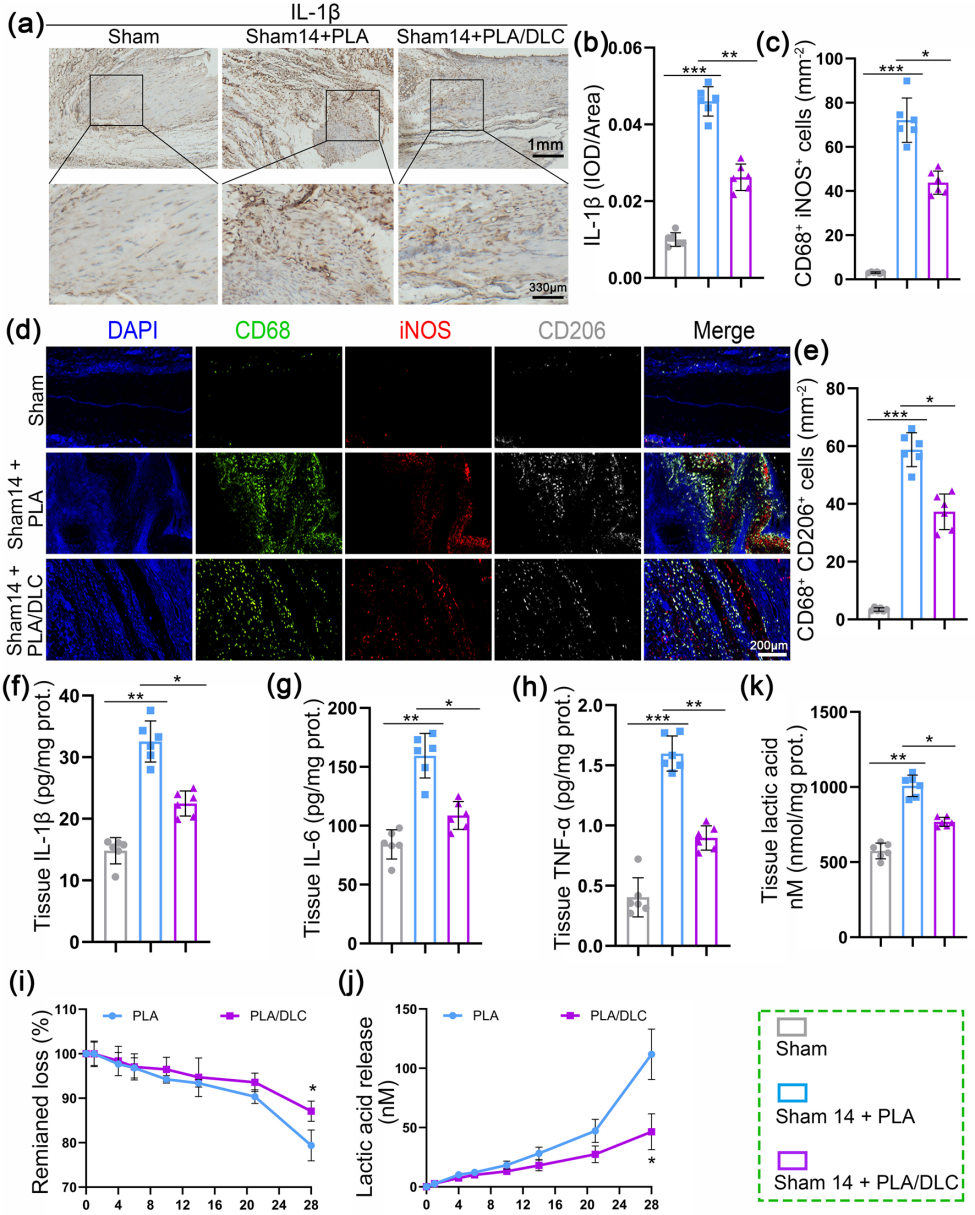
DLC inhibited PLA implantation-induced foreign body inflammation response and delayed PLA degradation. **a** Immunohistochemical staining for IL-1*β* in Sham group, Sham + PLA group, and Sham + PLA/DLC group at 14 d. **b** The optical density of IL-1*β* in the peritendinous tissues was measured. **c** The mean cell area of iNOS-positive cells in macrophages. **d** CD68/iNOS/CD206 fluorescence trichrome staining was used to measure the macrophage polarization around normal tendons. **e** The mean cell area of CD206-positive cells in macrophages. **f**–**h** The expression of IL-1β, IL-6, and TNF-*α* in normal peritendinous tissue was measured by ELISA after 14 d of implantation. **i** The remaining loss and **j** lactic acid release from enzymatic ester hydrolysis of PLA and PLA/DLC were measured at different time points. **k** The lactic acid level in the peritendinous tissue was measured in each group after 14 d of implanting PLA and PLA/DLC into the normal tendon. Data represent independent experiments, and all data are presented as mean ± SD; *NS* non-significant, *P* > 0.05; *P < 0.05; **P < 0.01; ***P < 0.001, ****P < 0.0001

**Fig. 7:**
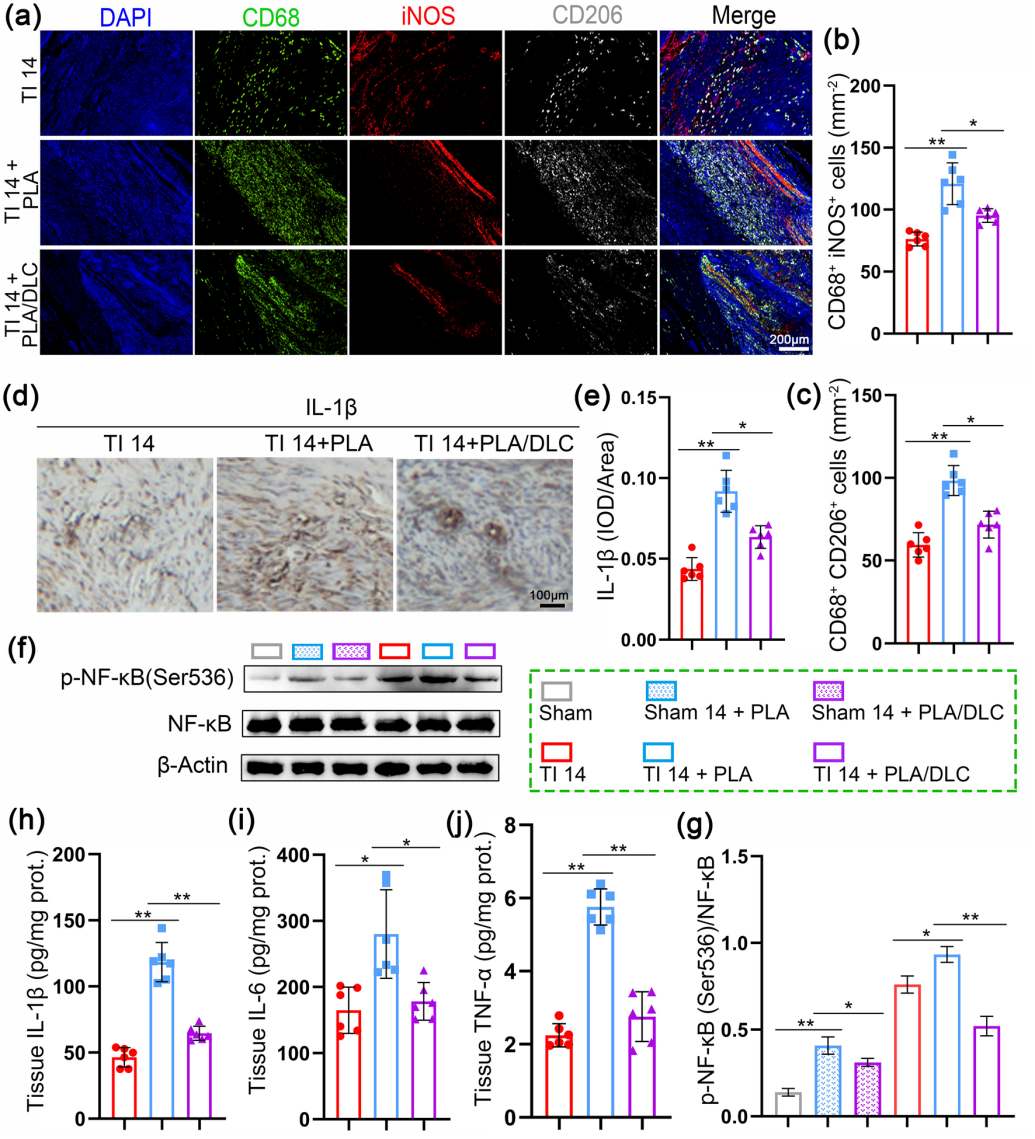
DLC suppressed the inflammatory response during tendon repair. **a** CD68/iNOS/CD206 fluorescence trichrome staining was used to measure the macrophage polarization around injured tendons. **b**, **c** The mean cell area of iNOS-positive and CD206-positive cells. **d**, **e** Immunohistochemical staining for IL-1*β* in the TI group, TI + PLA group, and TI + PLA/DLC group at 14 d, and relative IL-1*β*-positive cell area. **f** Representative bands of p-NF-κB in peritendinous tissues were measured by Western blotting. **h**–**j** The expression of IL-1*β*, IL-6, and TNF-*α* in peritendinous adhesion tissue of 14 d was measured by ELISA. **g** p-NF-κB expression was normalized to NF-κB expression. Data represent independent experiments, and all data are presented as mean ± SD; *NS* non-significant, *P* > 0.05; *P < 0.05; **P < 0.01; ***P < 0.001, ****P < 0.0001

Considering the inhibitory effect of DLC on PLA-implantation caused FBR, we evaluated peritendinous tissue inflammation level after PLA and PLA/DLC membrane implanted around the intact tendon in rats for 14 days (Fig. 5a). Immunohistochemical staining of IL-1*β* showed that the protein expression of IL-1*β* was significantly increased in the peritendinous tissue after PLA membrane implantation, which was effectively reversed after PLA/DLC membrane implantation (Fig. 6a, b). Moreover, DLC coating significantly reduced IL-6, IL-1*β*, and TNF-*α* levels in peritendinous tissues, which increased by PLA membrane implantation (Fig. 6f-h). We measured macrophage activation by immunofluorescence staining with CD68, iNOS, and CD206. As shown in Fig. 6c-e, PLA membrane implantation markedly increased the abundance of macrophages (CD68^+^ cells), and the number of macrophages in the PLA/DLC -implanted group was less than that in the PLA-implanted group. In addition, the number of CD68^+^iNOS^+^ cells and CD68^+^CD206^+^ cells in the PLA/DLC group were significantly less than in the PLA group. These results confirmed that DLC coating prevented NF-κB hyperphosphorylation at Ser536 in peritendinous tissues after PLA membrane implantation (Figs. 7f, g and S7). These results indicate that PLA membrane implantation induced foreign body inflammation, leading to M1 polarization of macrophages and promoting the expression of pro-inflammatory cytokines IL-6, IL-1β, and TNF-α. DLC coating also effectively inhibited PLA implantation-induced foreign body inflammation response.

The implantation of PLA membrane into the body leads to the development of an inflammatory and fibrotic process-FBR, thus limiting its applications in the clinic. The ROS regulates the process of FBR at the inflammation stage after the implantation of foreign material [42]. The DLC, which has great potential for biomedical applications, has been well acknowledged [46, 63, 64], and its ROS scavenging ability has also been identified above. In this study, the ROS scavenging ability of DLC further alleviated FBR caused by PLA membrane implantation (Fig. 5) by suppressing M1 polarization of macrophages and downregulating pro-inflammatory cytokines TNF-*α*, IL-6, and IL-1*β* (Figs. 4 and 6). In the process of peritendinous adhesion formation, NF-κB phosphorylation enhanced the M1 polarization of macrophages in response to the PLA membrane [17]. DLC reduced NF-κB hyperphosphorylation in the peritendinous adhesion tissue in our study (Fig. 7), which was consistent with previous findings that DLC can downregulate TNF-*α* and IL-6 via NF-κB and STAT3 signaling pathways [52]. Reduced NF-κB phosphorylation confirmed that DLC effectively mitigated FBR. Considering the cell signaling mechanisms involved in the pathogenesis of peritendinous adhesion formation, macrophages are the primary immune cells modulating the behavior of fibroblasts and myofibroblasts [65]. M1 polarized macrophages represent the pro-inflammatory phenotype of macrophages. They express pro-inflammatory cytokines and iNOS [66] in response to PLA implantation [17] and provoke the inflammatory response in the inflammation stage of peritendinous adhesion formation [67]. Besides acting on the FBR, DLC also inhibits NF-κB phosphorylation after tendon injury (Fig. 2), suggesting an anti-adhesion potential of DLC. To our knowledge, this is the first report showing that PLA modification by DLC coating attenuates PLA implantation-associated FBR, reducing the side effects of PLA membrane and expanding its application.

### DLC Depositing Delayed the Degradation of the PLA Membrane

The degradation of PLA membrane and PLA/DLC membrane was first assessed in vitro by measuring the percentage of remaining loss and the concentration of lactic acid at each time point. There was a noticeable difference in the remaining loss of degraded membranes between the PLA membrane and PLA/DLC membrane on the 28th day (Fig. 6i). Consistent with membrane degradation, the lactic acid released from the PLA/DLC membrane significantly decreased on the 28th day compared to that released from the PLA membrane (Fig. 6j). These results suggest that DLC coating delayed PLA degradation. No material was implanted around the tendon in the sham group. Consistent with in vitro results, PLA membrane implantation significantly elevated the lactic acid level in peritendinous tissues (Fig. 6k), indicating obvious degradation of PLA membrane and lactic acid release. Moreover, the lactic acid level in the PLA/DLC group was markedly lower than in the PLA group (Fig. 6k), suggesting that DLC delayed PLA degradation in vivo. Similarly, the PLA/DLC group also displayed significantly lower lactic acid content in peritendinous adhesion than the PLA group when the membranes were implanted around the injured tendon (Fig. S8). These results suggested that DLC coating obviously delayed the PLA membrane degradation.

### DLC Alleviated the Inflammatory Response during Tendon Repair

Inflammation and macrophages recruited mesenchymal stromal cells (MSCs) to form myofibroblasts in peritendinous adhesions [67]. Thus, we evaluated the anti-inflammatory potential of DLC in a rat model of Achilles tendon adhesion 14 days after tendon injury. After tendon injury, there was a substantial presence of macrophages, including CD68^+^iNOS^+^ cells and CD68^+^CD206^+^ cells in peritendinous tissues, with PLA membrane implantation significantly increased the number of these macrophages. The number of CD68^+^iNOS^+^ cells and CD68^+^CD206^+^ cells markedly decreased in the PLA/DLC group compared to the PLA group (Fig. 7a-c). Correspondingly, DLC coating significantly reduced the increased expression of IL-1*β* in peritendinous tissue after PLA membrane implantation on the injured tendon (Fig. 7d, e). In addition, DLC coating significantly reduced the increased levels of IL-6, IL-1*β*, and TNF-*α* in peritendinous tissue after PLA membrane implantation (Fig. 7h-j). Moreover, DLC coating reversed NF-κB hyperphosphorylation at Ser536 in peritendinous tissues (Figs. 7f, g and S7). DLC effectively suppressed the inflammatory response following PLA implantation on the injured tendon.

M2 (polarized macrophages) also played a significant role in the pathogenesis of peritendinous adhesion formation. M2 macrophages are the regenerative macrophages that highly express CD206. They respond to PLA biodegradation and lactic acid production [11] and participate in cell proliferation and matrix deposition [68, 69] at the proliferation stage of peritendinous adhesion formation. By secreting TGF-*β*1, they recruit mesenchymal stromal cells to form myofibroblasts [67]. From this perspective, our study revealed that DLC coating significantly inhibited PLA biodegradation-mediated increase in CD68^+^CD206^+^ cell abundance (Fig. 7) and then decreased the content of TGF-*β*1 (Fig. S9) in the proliferation stage (14 days) of tendon injury, indicating an anti-adhesion capacity of DLC. The implanted PLA membrane in the body could be degraded by esterase, and then, the degradation products lactic acid and glycolic acid were released, increasing the local acidic toxicity [11]. When the PLA fibers compactly deposited with DLC films by the PECVD method (Fig. 1), DLC films blocked the ester bonds in PLA fibers from esterase spatially. Thus, PLA/DLC displayed delayed degradation rate in vitro and in vivo (Fig. 6i-k). In addition, long-term implantation may lead to the formation of small particles during tendon gliding, inducing joint pain, osteolysis, or implant loosening [70] and ultimately undermining the anti-adhesion property. DLC coating has been revealed to enhance wear resistance and prevent metal ion release [64, 71]. In our study, DLC coating effectively restricted lactic acid release (Figs. 6i-k and S8) to surrounding tissues and decreased downstream M2 macrophages (Figs. 6 and 7).

### DLC Depositing Improved the Anti-Adhesion Effect of the PLA Membrane

**Fig. 8:**
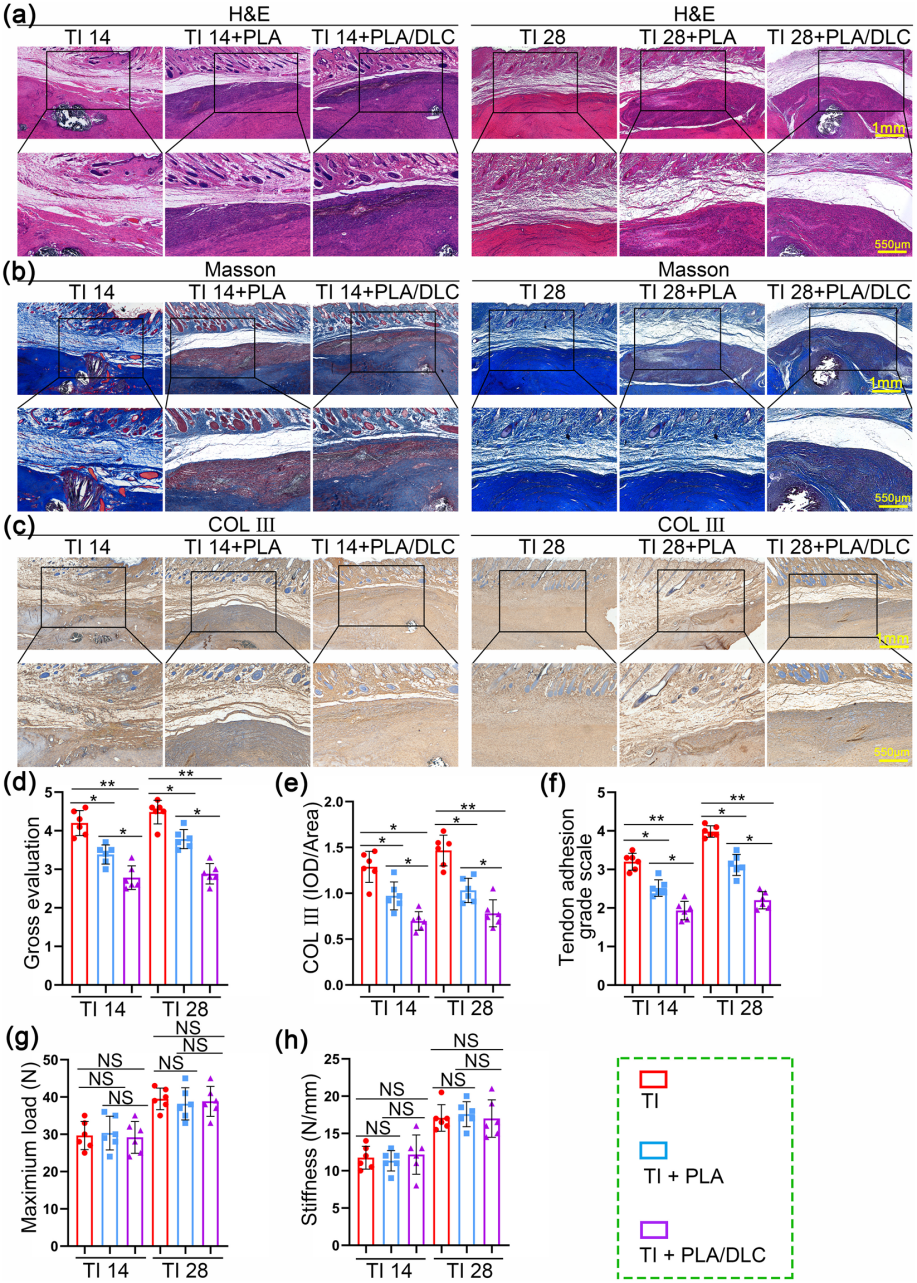
DLC depositing improved the anti-adhesion efficiency of the PLA membrane. **a** H&E staining and **b** Masson trichrome staining of normal tendon wrapped by PLA and PLA/DLC for 14 and 28 days. **c** Immunohistochemical staining for COL III of TI group, TI + PLA group, and TI + PLA/DLC group at 14 and 28 days. **d** Gross histological view, **e** Average optical density of COL III in the peritendinous adhesion tissues were exhibited. **f** Adhesion score was assessed after 14 and 28 days. **g** Maximum load and **h** stiffness of the regenerated tendon. Data represent independent experiments, and all data are presented as mean ± SD; *NS* non-significant, *P* > 0.05; *P < 0.05; **P < 0.01; ***P < 0.001, ****P < 0.0001)

Due to the observed antioxidant and anti-inflammatory activities of the PLA/DLC membrane, we assessed the anti-adhesion efficiency of PLA/DLC on the rat model of peritendinous adhesion on the 14th day and 28th postoperative days. Rats were subjected to the Achilles adhesion model in the tendon injury (TI) group and did not undergo membrane implantation. The gross view is shown in Fig. S10 and Video S1. No visible inflammation hallmarks or infective ulcers were observed in gross observation. At 14 days and 28 days, the dense adhesive tissues were observed around the injured sites and were difficult to separate in the TI group bluntly. Adhesive tissues were also observed in the PLA group but could be separated with a blunt instrument. Almost no obvious tissues were observed on the repaired tendon in the PLA/DLC group. The gross grading was quantified using the gross adhesion scoring system (Fig. S11 and Video S2). As shown in Fig. 8d, the scores of the PLA and PLA/DLC groups were significantly lower than those of the TI group. The score of the PLA/DLC group was also markedly lower than that of the PLA group. These results clarified the greater anti-adhesion efficiency of the PLA/DLC membrane than the PLA membrane.

H&E staining and Masson staining were performed, and the adhesion score system (Fig. S12) was used to assess adhesion tissue formation in rats, and a higher score means more severe peritendinous adhesion formation. As displayed in Fig. 8a, b, and f, intense fibrous tissue formation was observed in the peritendinous area of the TI group at 14 days and 28 days. That is to say, the structural tendon injury was repaired by the adhesion tissues as a process of exogenous healing. However, in the TI group the tendon gliding was limited due to the adhesion formation. Thereafter, the adhesion formation is a non-regenerative healing process. Among them, adhesive tissue formation was milder in both PLA and PLA/DLC groups than in the TI group, and the adhesion scores of the PLA/DLC group were significantly lower than that of the PLA group at 14 days and 28 days. The none-regenerative healing process was modulated in both PLA and PLA/DLC groups. DLC coating significantly elevated the anti-adhesion efficiency of PLA membrane by 23.41% and 29.49% at 14 days and 28 days, respectively. Moreover, PLA/DLC group also displayed fewer myofibroblasts in peritendinous adhesion tissue (Fig. S13). In addition, immunohistochemical and Sirius red staining were performed to assess the expression of COL III in peritendinous adhesive tissue (Figs. 8c, e and S14). The expression of COL III in the PLA/DLC group was significantly lower than that in the PLA group at 14 days and 28 days, suggesting that the PLA/DLC membrane possessed greater anti-adhesion capacity than the PLA membrane. The strength of tendon healing, including the maximum load and stiffness, was measured for mechanical analysis (Fig. S15). The maximum load and stiffness of the three groups were not statistically different at 14 and 28 days (Fig. 8g, h), indicating that PLA and PLA/DLC membranes had no significant effect on tendon healing after injury. All these results demonstrated that PLA/DLC membrane inhibited peritendinous adhesion formation after tendon injury, and DLC coating improved the anti-adhesion efficiency of the PLA membrane.

Despite showing DLC's antioxidant, anti-inflammatory, and anti-biodegradation capacity in preventing peritendinous adhesion formation, our study has some limitations. The specific relationships or mechanisms between surface functional groups and chemical bond of DLC and displayed ROS scavenging ability and anti-inflammatory property needs further investigation. Moreover, further studies are needed to take advantage of DLC for drug, gene, and peptide delivery and prevent adhesion formation. DLC can help efficient anti-adhesion barrier membranes with fewer side effects.

## Conclusion

**Fig. 9:**
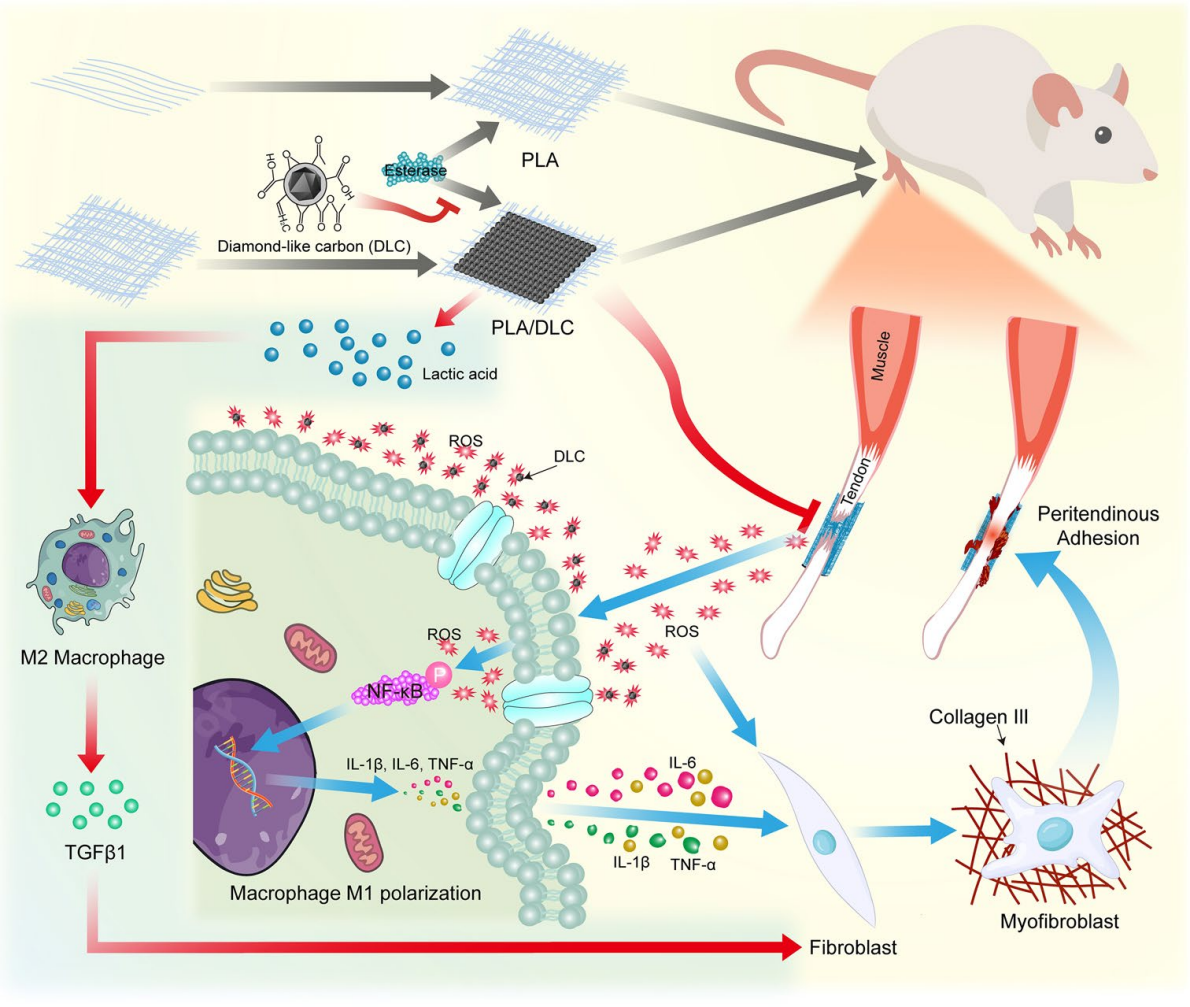
Putative mechanisms by which DLC-deposited PLA membrane displays more effective anti-adhesion capacity than the PLA membrane. DLC reduces the production of ROS and alleviates oxidative stress because of the abundant functional groups on the DLC surface. Decreasing ROS levels suppresses NF-κB phosphorylation and then mitigates macrophage M1 polarization during FBR after PLA implantation. DLC blocks the ester bonds in PLA fibers from esterase spatially and delay lactic acid releasing, thereby inhibiting the lactic acid-induced M2 polarization of macrophages. Thus, DLC reduces the side effects of the implants and enhance their anti-adhesion efficiency

DLC-deposited PLA membrane displayed more effective anti-adhesion capacity than the PLA membrane. DLC displayed ROS scavenging property because of the abundant C=O dangling bonds on the surface. On the one hand, DLC reduced the production of ROS and alleviated oxidative stress following tendon injury at the inflammation stage of peritendinous adhesion formation. The ROS scavenging ability of DLC also mitigated macrophage M1 polarization-mediated inflammation through reduced NF-κB phosphorylation during the peritendinous adhesion formation after tendon injury, as well as in FBR after PLA implantation. On the other hand, DLC delayed PLA biodegradation and lactic acid release to surrounding tissue, thereby inhibiting the lactic acid-induced M2 polarization of macrophages at the proliferation stage of peritendinous adhesion formation (Fig. 9), leading to prolonged separate effects and enhanced anti-adhesion potential. Thus, DLC depositing on PLA possesses an efficient biophysical mechanism for the prevention of peritendinous adhesion.

## Supplementary Information

Below is the link to the electronic supplementary material.

Supplementary file1 (MP4 15250 kb)

Supplementary file2 (MP4 13288 kb)

Supplementary file3 (DOCX 6252 kb)
