# Supplementary material for: Diamond-Like Carbon Depositing on the Surface of Polylactide Membrane for Prevention of Adhesion Formation During Tendon Repair
Source: Nanomicro Lett. 2024 Apr 30;16:186. doi: 10.1007/s40820-024-01392-7 (PMC11061095; doi:10.1007/s40820-024-01392-7)
Supplement: Supplementary file 3 — Supplementary file3 (DOCX 6252 kb) [file 40820_2024_1392_MOESM3_ESM.docx]

Supporting Information for

**Diamond-Like Carbon Depositing on the Surface of Polylactide Membrane for Prevention of Adhesion Formation during Tendon Repair**

Yao Xiao^1, #^, Zaijin Tao^1, #^, Yufeng Ju^2, #^, Xiaolu Huang^3^, Xinshu Zhang^1^, Xiaonan Liu^1^, Pavel А. Volotovski^4^, Chao Huang^5^, Hongqi Chen^6,^ *, Yaozhong Zhang^7,^ * and Shen Liu^1,^ *

^1^Department of Orthopaedics, Shanghai Jiao Tong University School of Medicine Affiliated Sixth People’s Hospital, 600 Yishan Rd, Shanghai, 200233, P. R. China

^2^ Shanghai Tongji Hospital, 389 Xincun Rd, Shanghai, 200065, P. R. China

^3^Key Laboratory for Thin Film and Microfabrication of Ministry of Education, Research Institute of Micro/Nano Science and Technology, Shanghai Jiao Tong University, Shanghai, 200240, P. R. China

^4^Orthopedic Trauma Department, Belarus Republic Scientific and Practical Center for Traumatology and Orthopedics, Kizhevatova str., 60/4, Minsk, 220024, Belarus

^5^Shanghai Haohai Biological Technology Limited Liability Company, 1386 Hongqiao Rd, Shanghai, 200336, P. R. China

^6^Department of General Surgery, Shanghai Jiao Tong University School of Medicine Affiliated Sixth People’s Hospital, 600 Yishan Rd, Shanghai, 200233, P. R. China

^7^Shanghai Key Laboratory for High Temperature Materials and Precision Forming, School of Materials Science and Engineering, Shanghai Jiao Tong University, Shanghai, 200240, P. R. China.

^#^ Yao Xiao, Zaijin Tao, and Yufeng Ju contributed equally to this work.

*Corresponding authors. E-mail: [liushensjtu@sjtu.edu.cn](mailto:liushensjtu@sjtu.edu.cn) (Shen Liu); [zhangragnar@sjtu.edu.cn](mailto:zhangragnar@sjtu.edu.cn) (Yaozhong Zhang); [hqchen08@163.com](mailto:hqchen08@163.com) (Hongqi Chen)

**Supplementary Figures and Table**

**
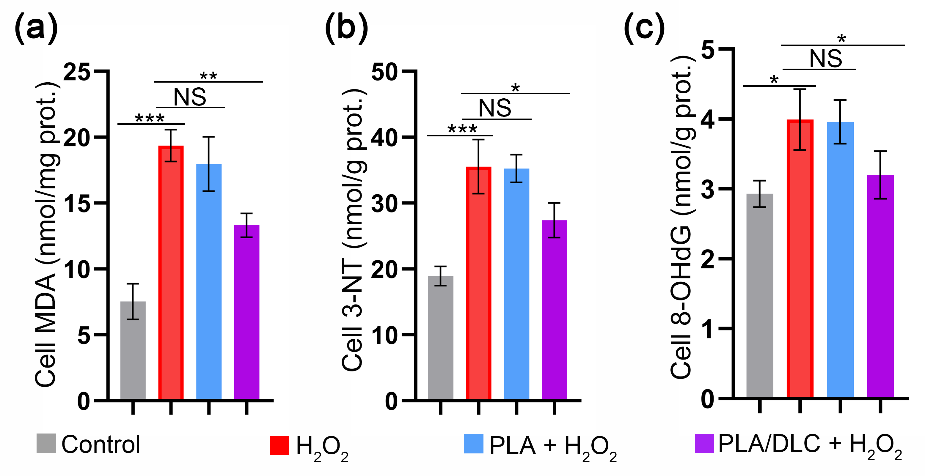
**

**Fig. S1** **The scanning electron microscope image of PLA and PLA/DLC membrane.** Content of MDA (**a**), 3-NT (**b**), and 8-OHdG (**c**) in H_2_O_2_-induced NIN/3T3 cells was examined by assay kit respectively

**
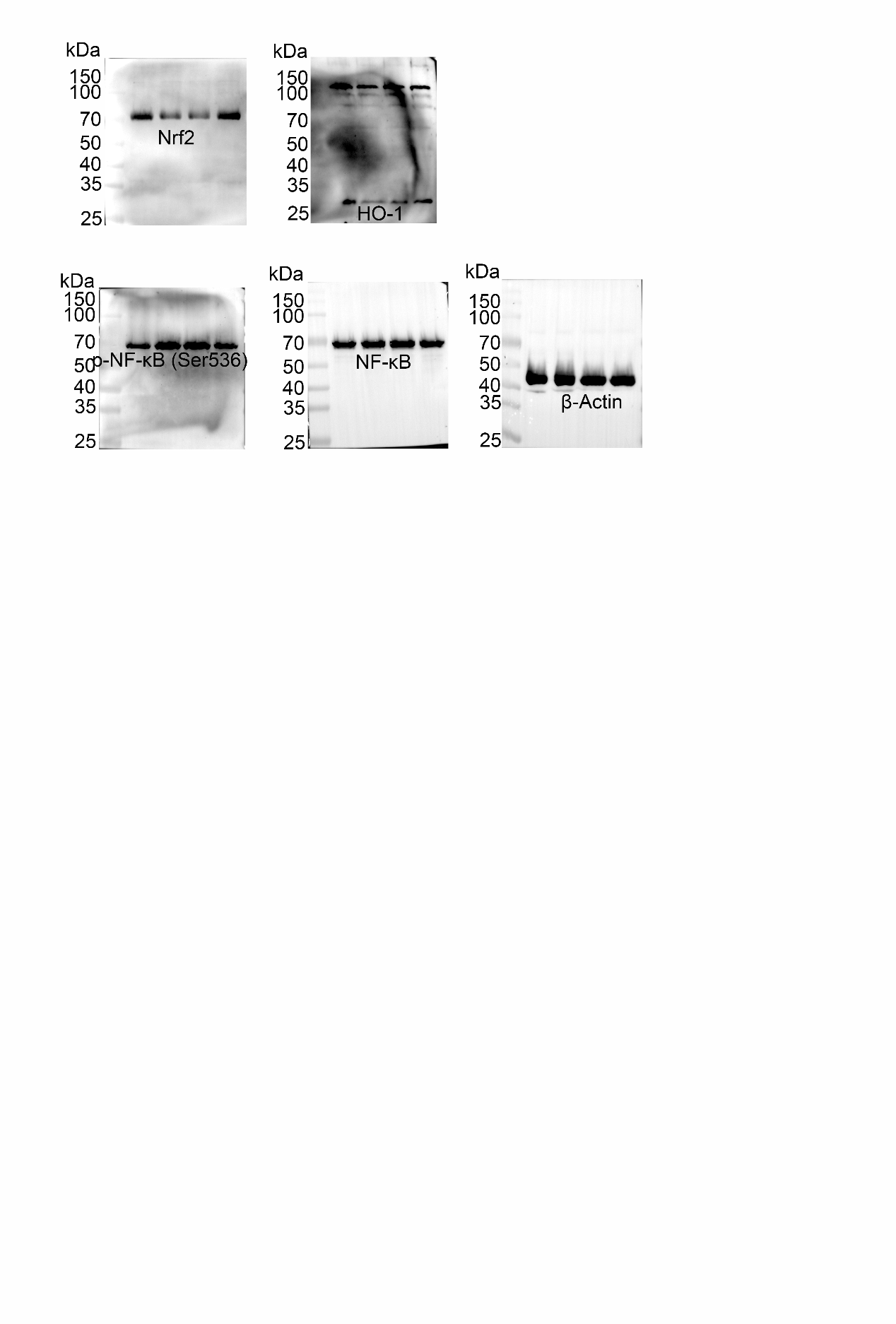
**

**Fig. S2** The western bolt analysis of peritendinous tissues follow membrane implant for 7 days after tendon injury


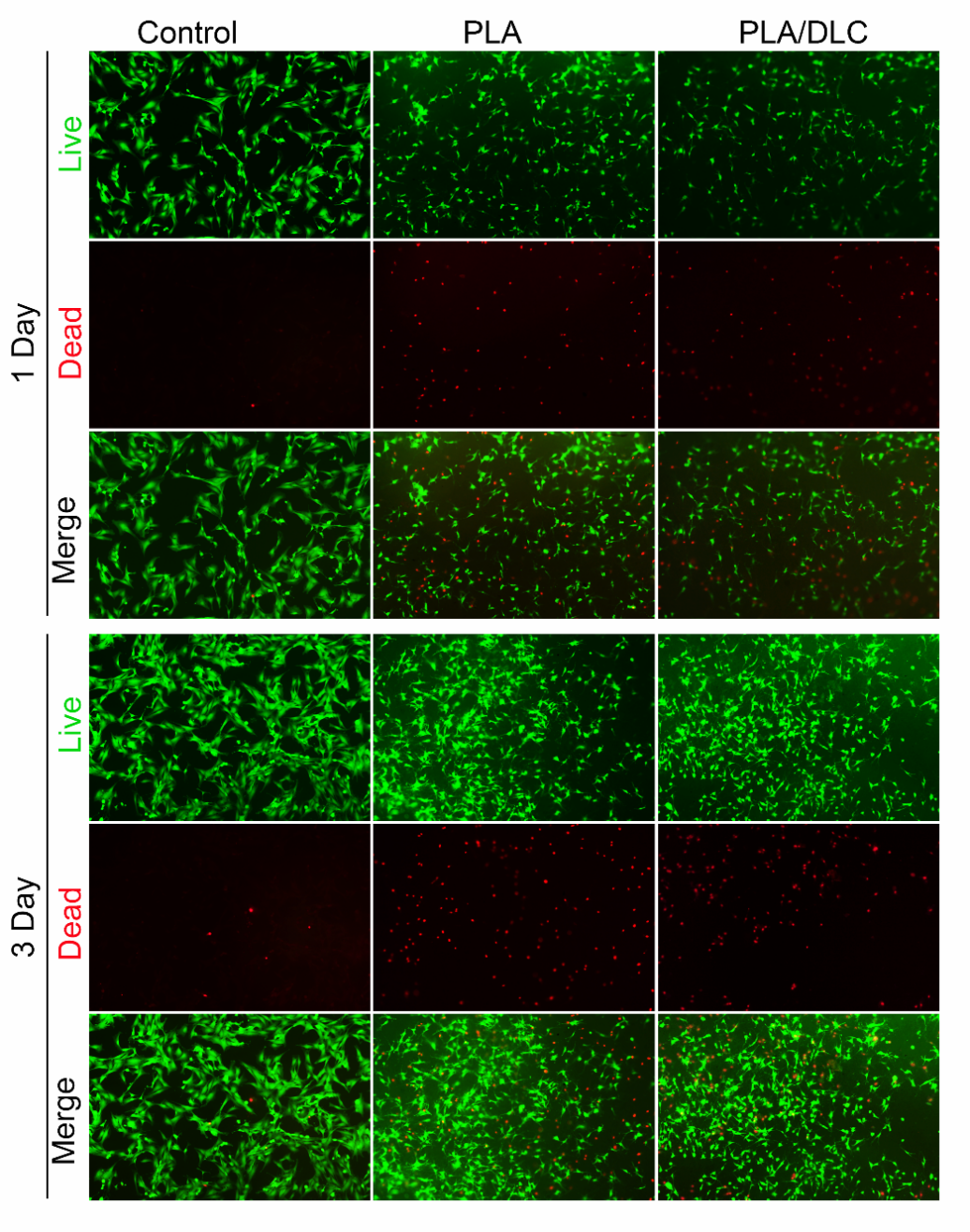


**Fig. S3** Live/dead staining of fibroblasts cultured on different membranes for 1 and 3 days


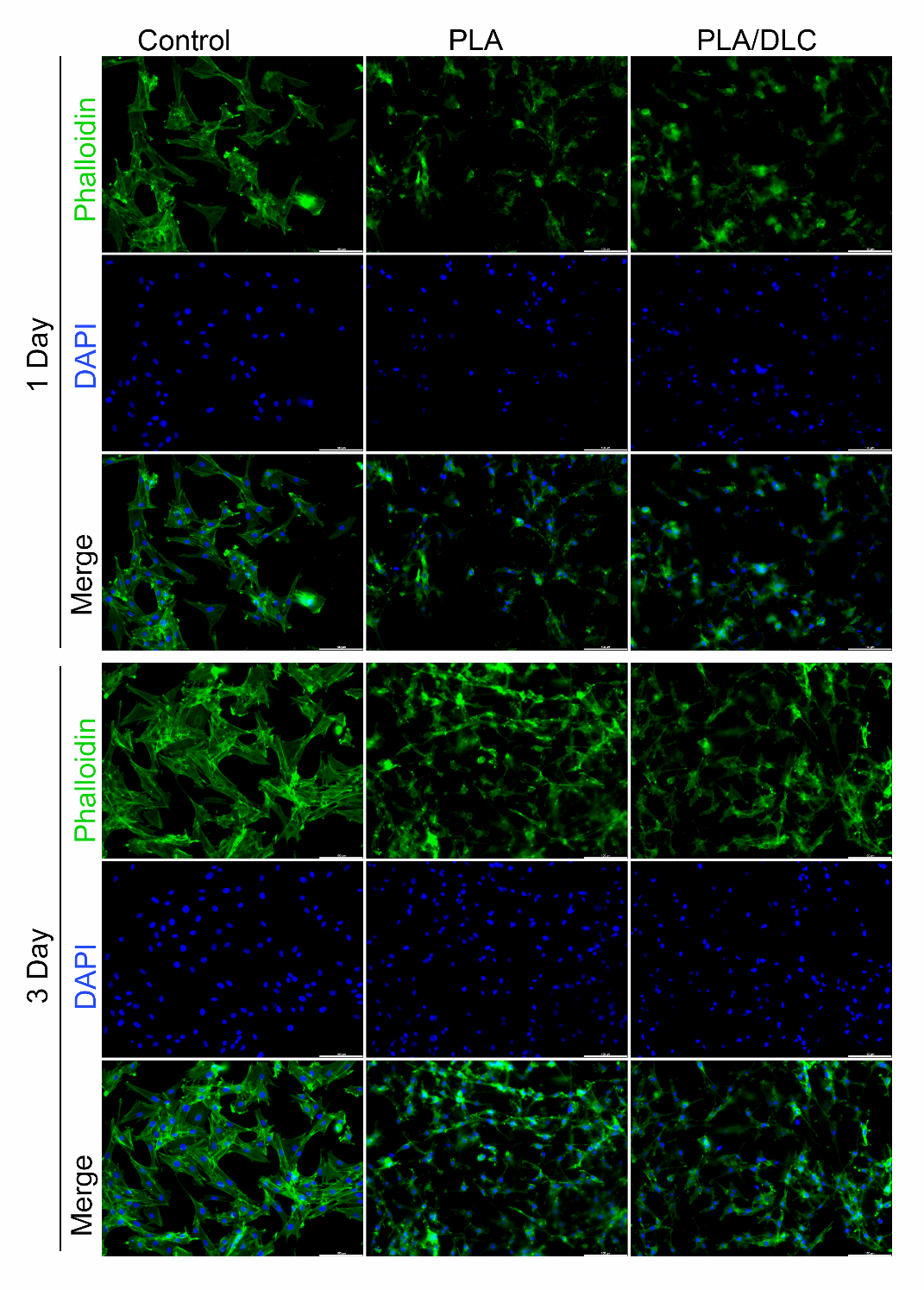


**Fig. S4** Phalloidin staining for adhesion analysis of fibroblasts cultured on different membranes at days 1 and 3

**
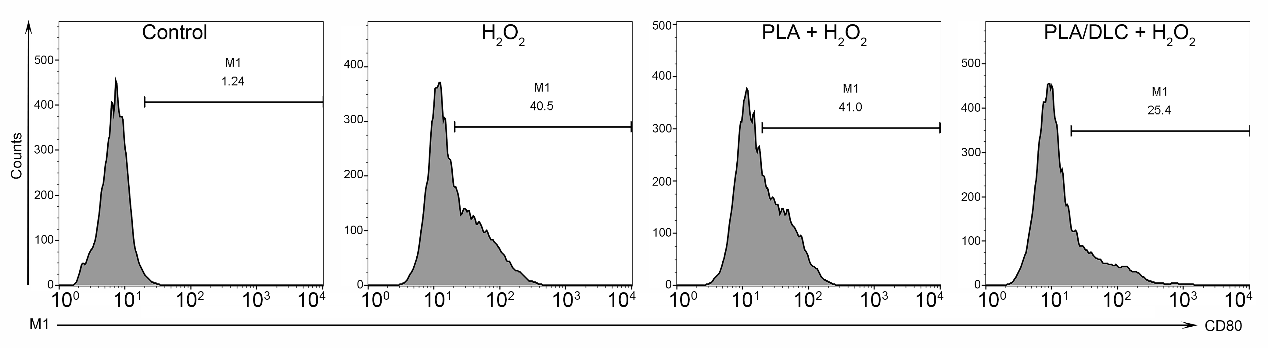
**

**Fig. S5** Flow cytometry results of macrophage M1 polarization


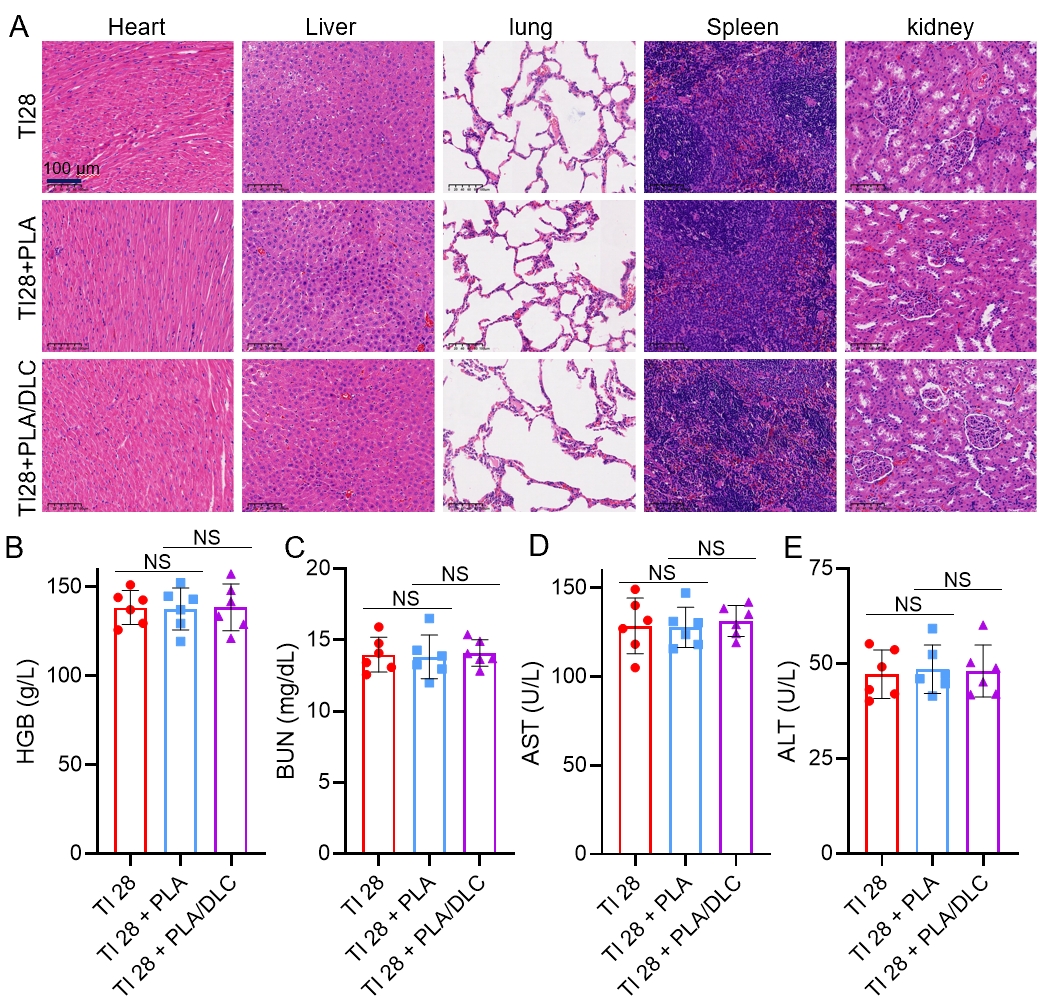


**Fig. S6 Biocompatibility and biosafety evaluation.** (**a**) H&E staining and (**b-e**) blood routine and biochemical analysis. Data represent independent experiments, and all data are presented as mean ± SD; NS: non-significant. HGB: haemoglobin. BUN: blood urea nitrogen. AST: aspartate aminotransferase. ALT: alanine aminotransferase

**
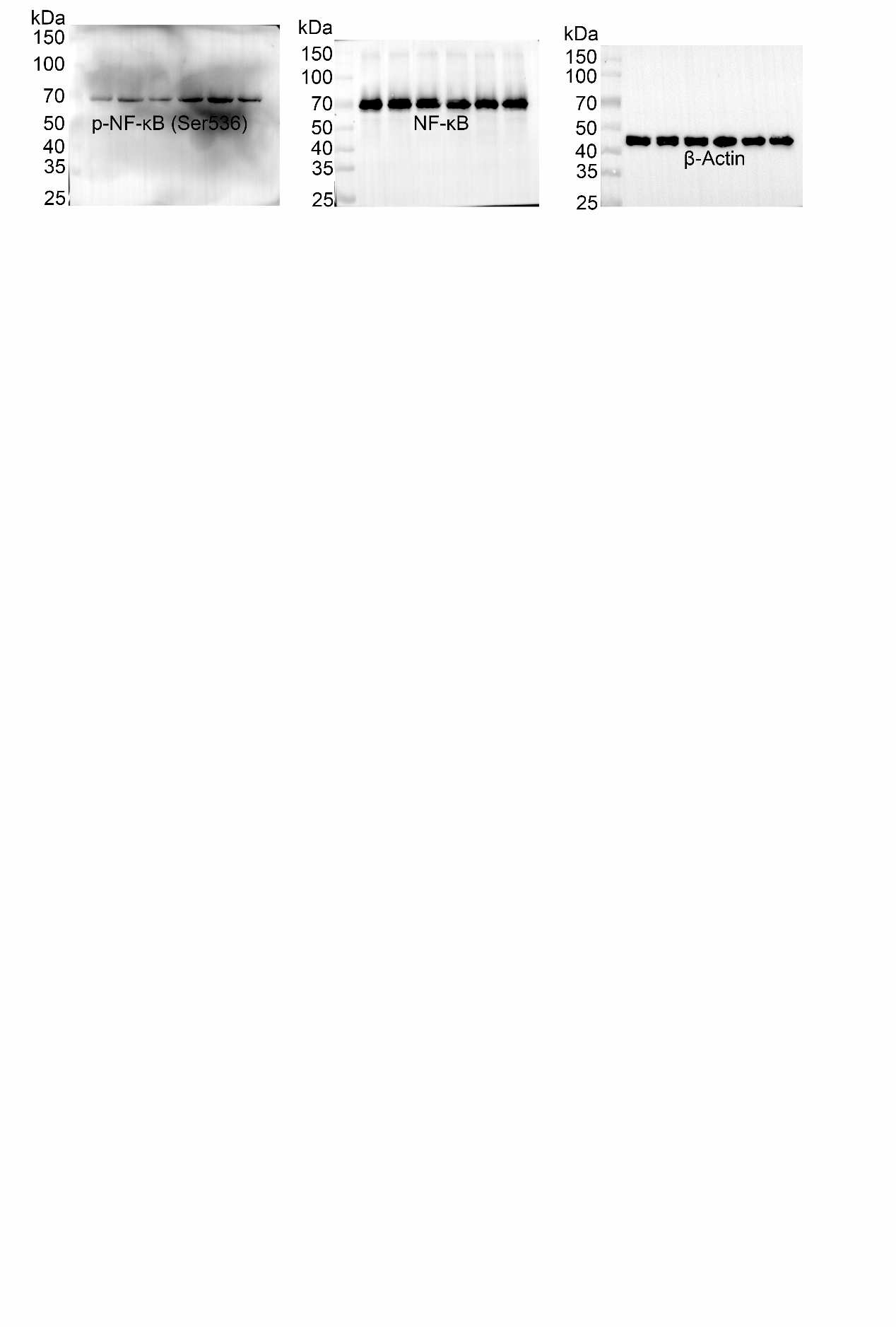
**

**Fig. S7** The western bolt analysis of peritendinous tissues follow membrane implant for 14 days


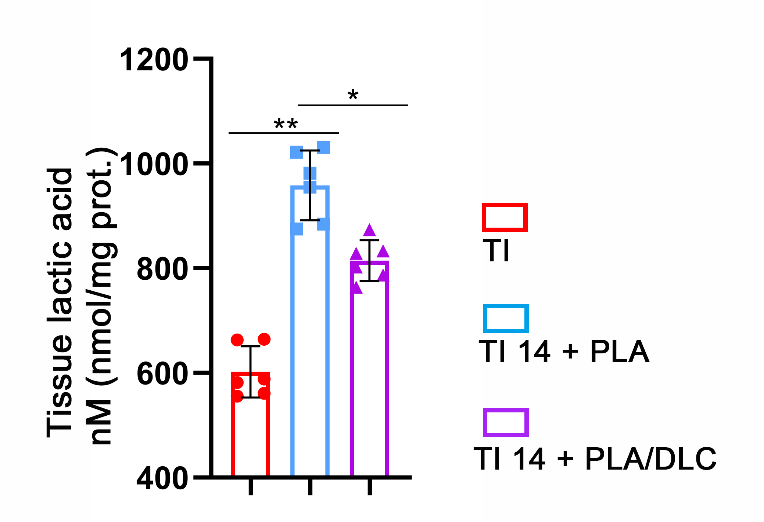


**Fig. S8** The lactic acid content in the peritendinous adhesion tissues following membrane implant for 14 days after tendon injury


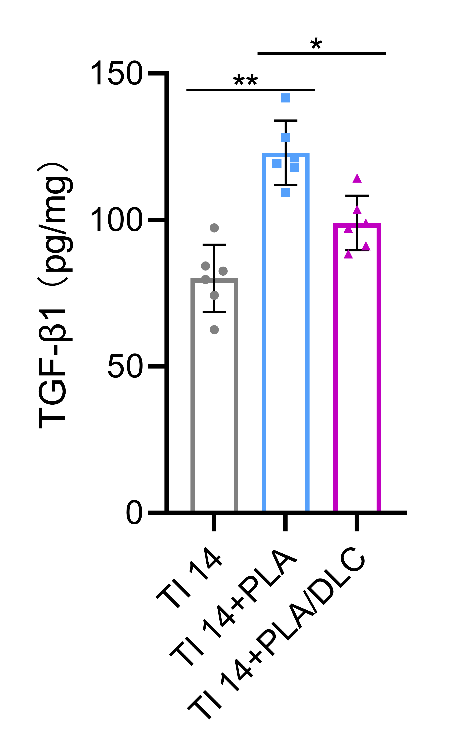


**Fig. S9** The TGF-β1 content in the peritendinous adhesion tissues following membrane implant for 14 days after tendon injury

**
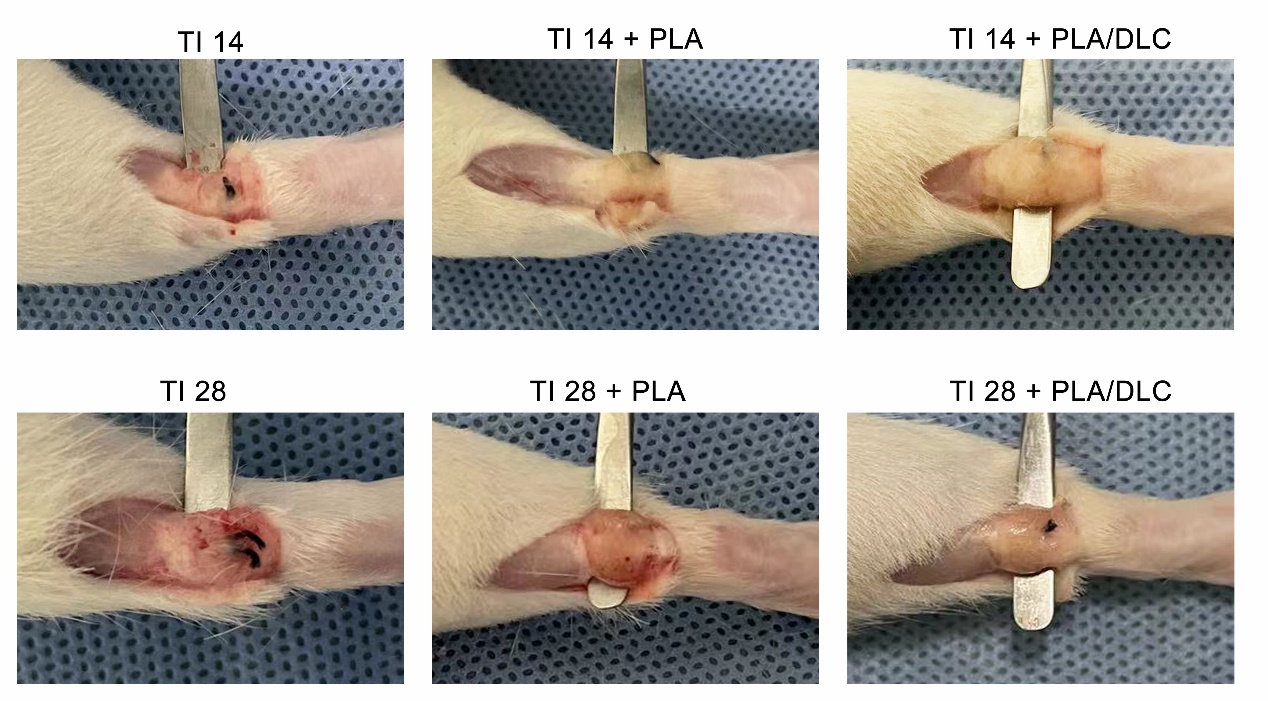
**

**Fig. S10** The Gross observation of peritendinous tissues after tendon injury and membrane implantation for 14 and 28 days


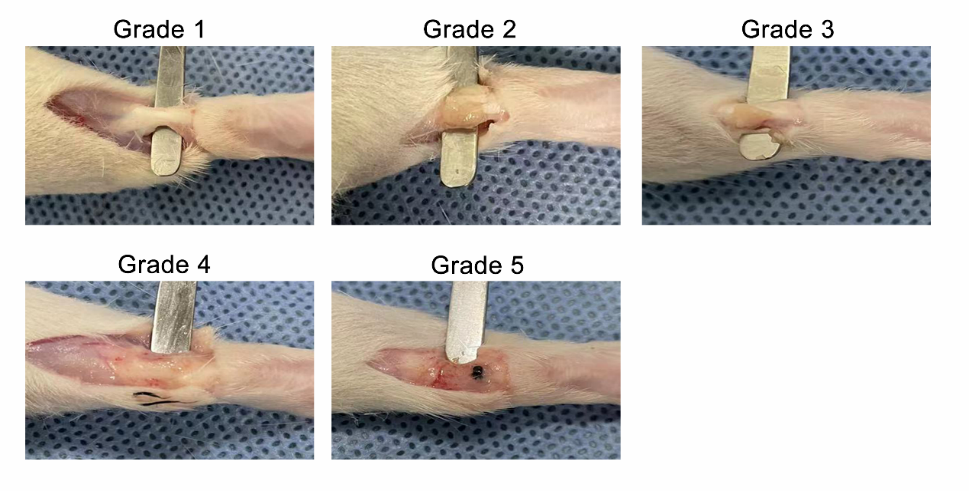


**Fig. S11 The gross evaluation standards for peritendinous adhesion.** The adhesion scoring was classified into 5 grades: 1, no obvious adhesion at surgical site; 2, a little of adhesion tissue but can be bluntly separated; 3, less than 50% of the adhesion tissue that needed sharp separation; 4, about 51% - 97.5% of the adhesion tissue which needed sharp separation; 5, more than 97.5% severe adhesion tissue that required sharp separation


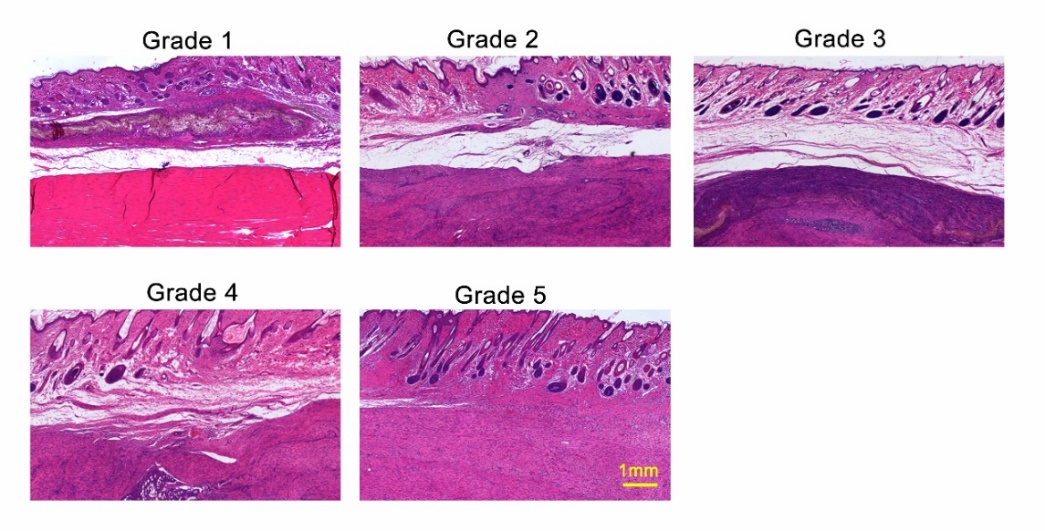


**Fig. S12 The histological evaluation standards for peritendinous adhesion.** Grade 1, no adhesions in the peritendinous area of the repaired site; Grade 2, adhesions possessing less than 25% in the peritendinous area of the repaired site; Grade 3, adhesions possessing 25%~50% in the peritendinous area of the repaired site; Grade 4, adhesions possessing 50%~75% in the peritendinous area of the repaired site; Grade 5, adhesions constituting more than 75% of the area of the repaired site


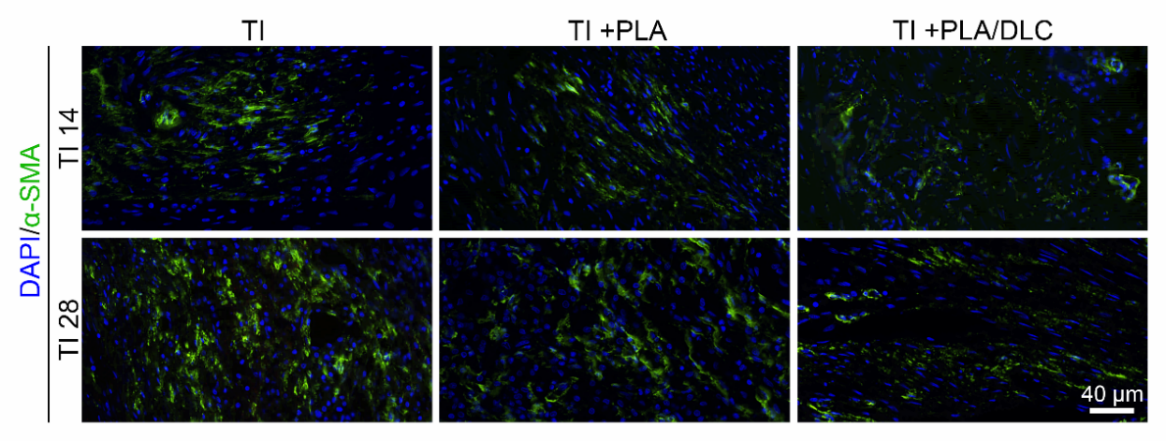


**Fig. S13** The myofibroblast (α-SMA) in peritendinous adhesion tissue after tendon injury


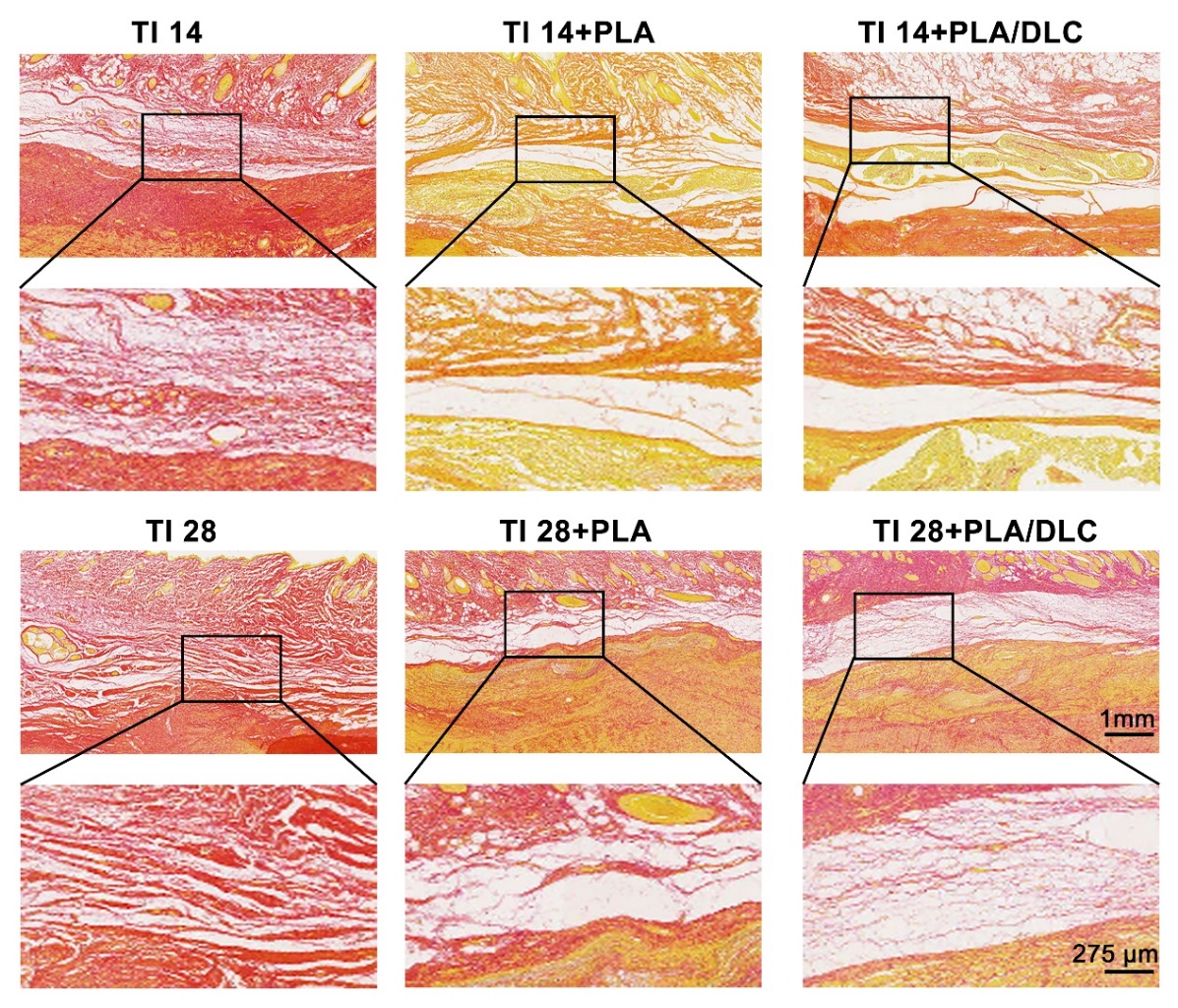


**Fig. S14** The Sirius red staining and polarized light observation of peritendinous adhesion tissue after tendon injury


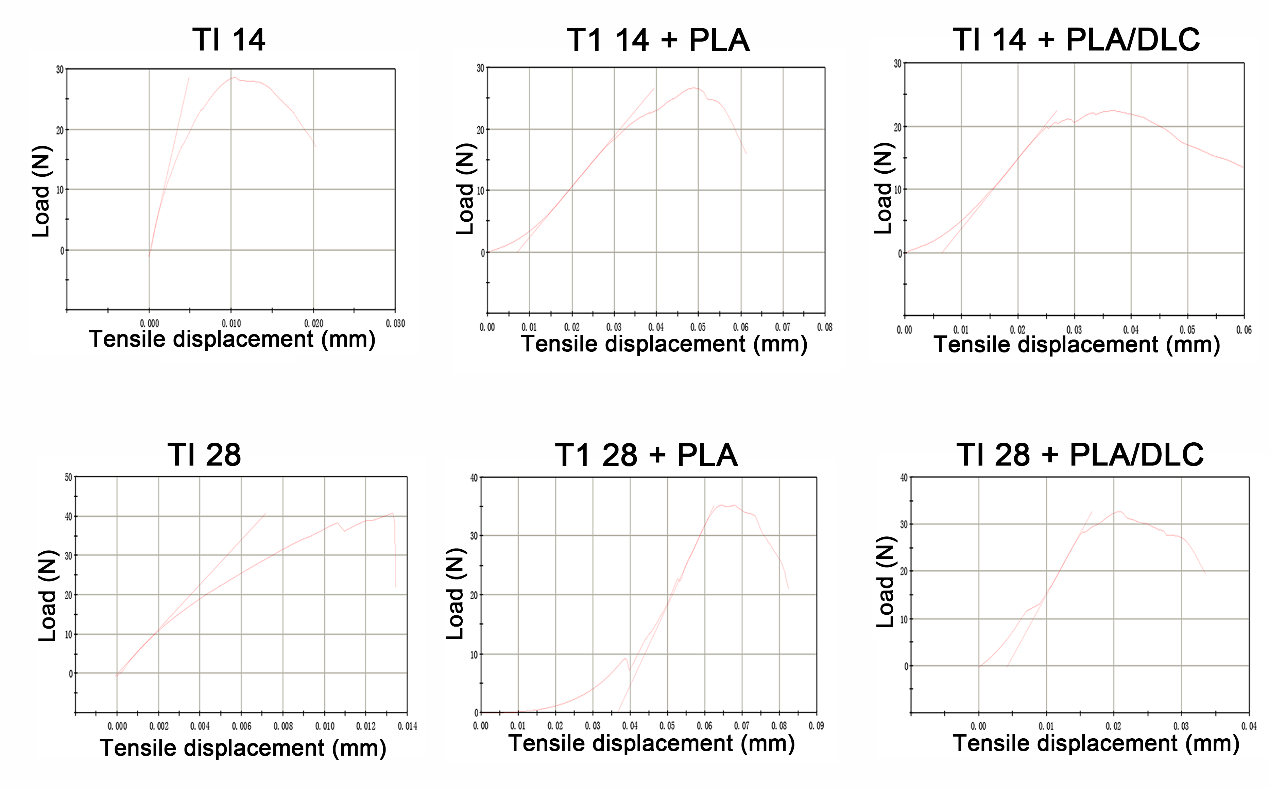


**Fig. S15** Maximal tendon breaking strength and stiffness of repaired tendons were measured using a rheometer until the tendons were disrupted.

**Table S1** Primer sequences for RT-PCR in this study

| Gene | Forward Sequence (5’-3’) | Reverse Sequence (5’-3’) |
| --- | --- | --- |
| Mouse Il-6 | AAGAAATGATGGATGCTACC | GAGTTTCTGTATCTCTCTGAAG |
| Mouse Tnf [1] | GGCACTCCCCCAAAAGATG | AGGAATGAGAAGAGGCTGAGACA |

**Supplementary Reference**

1. M. J. Charron, L. Williams, Y. Seki, X. Q. Du, B. Chaurasia, A. Saghatelian, S. A. Summers, E. B. Katz, P. M. Vuguin, S. E. Reznik. Antioxidant effects of n-acetylcysteine prevent programmed metabolic disease in mice. Diabetes. **69**(8), 1650-1661 (2020). <https://doi.org/10.2337/db19-1129>
